# iTRAQ-Based Quantitative Proteomic Comparison of 2D and 3D Adipocyte Cell Models Co-cultured with Macrophages Using Online 2D-nanoLC-ESI-MS/MS

**DOI:** 10.1038/s41598-019-53196-0

**Published:** 2019-11-14

**Authors:** Sun Young Lee, Sung Bum Park, Young Eun Kim, Hee Min Yoo, Jongki Hong, Kyoung-Jin Choi, Ki Young Kim, Dukjin Kang

**Affiliations:** 1 College of Pharmacy, Kyung Hee University, Seoul, 02447 Republic of Korea; 2 Therapeutics & Biotechnology Division, Korea Research Institute of Chemical Technology, 141 Gajeong-ro, Yuseong-gu, Daejeon 305-600 Republic of Korea; 3 Center for Bioanalysis, Division of Chemical and Medical metrology, Korea Research Institute of Standards and Science, Daejeon, 34113 Republic of Korea

**Keywords:** Mass spectrometry, Proteomic analysis

## Abstract

The demand for novel three-dimensional (3D) cell culture models of adipose tissue has been increasing, and proteomic investigations are important for determining the underlying causes of obesity, type II diabetes, and metabolic disorders. In this study, we performed global quantitative proteomic profiling of three 3D-cultured 3T3-L1 cells (preadipocytes, adipocytes and co-cultured adipocytes with macrophages) and their 2D-cultured counterparts using 2D-nanoLC-ESI-MS/MS with iTRAQ labelling. A total of 2,885 shared proteins from six types of adipose cells were identified and quantified in four replicates. Among them, 48 proteins involved in carbohydrate metabolism (e.g., PDHα, MDH1/2, FH) and the mitochondrial fatty acid beta oxidation pathway (e.g., VLCAD, ACADM, ECHDC1, ALDH6A1) were relatively up-regulated in the 3D co-culture model compared to those in 2D and 3D mono-cultured cells. Conversely, 12 proteins implicated in cellular component organisation (e.g., ANXA1, ANXA2) and the cell cycle (e.g., MCM family proteins) were down-regulated. These quantitative assessments showed that the 3D co-culture system of adipocytes and macrophages led to the development of insulin resistance, thereby providing a promising *in vitro* obesity model that is more equivalent to the *in vivo* conditions with respect to the mechanisms underpinning metabolic syndromes and the effect of new medical treatments for metabolic disorders.

## Introduction

Obesity is a major public health problem worldwide. It is one of numerous contributors to type II diabetes (T2D), hypertension, cancer, sleep-disordered breathing, insulin resistance and cardiovascular disease, which are characteristic of metabolic syndromes^1–4^. Obesity results from excess body fat that is caused by an increase in adipocyte volume (hypertrophy), adipocyte number (hyperplasia), or both (hypertrophy–hyperplasia)^5,6^. Adipocytes, which play key roles in the endocrine system, regulate the body’s metabolism through secreted cytokines^7–9^. Adipose-derived materials, such as cytokines, lipids and their derivatives, contribute to the development of insulin resistance^3^. Furthermore, the mitochondria of adipocytes produce reactive oxygen species (ROS), and their dysfunction is intimately associated with the occurrence of insulin resistance^10^.

Adipose cells are mainly classified into three types: white adipose tissue (WAT) cells, brown adipose tissue (BAT) cells, and brite (or beige) adipose tissue cells^11–13^. WAT cells play a vital role in the storage of energy and lipids known as triglycerides, as well as in the secretion of cytokines (or adipokines). BAT cells are involved in fat-burning for heat generation and energy expenditures that control energy homeostasis. Brite (or beige) adipose tissue cells share some characteristics with WAT and BAT. Subcutaneous white adipocytes were reported to convert to pink adipocytes in the mammary gland during pregnancy, lactation, and post-laction^14^. Adipose tissues are composed of a diverse population of cells, including adipocytes, preadipocytes, fibroblasts, endothelial cells, blood cells, macrophages, and multipotent stem cells. Adipose tissues represent a peculiar endocrine organ that secretes various hormones, such as adipokines^3,15,16^. Several studies have described how the infiltration of macrophages into human and murine adipose cells is closely connected to heavier body weights and the development of insulin resistance^17–21^.

In a two-dimensional (2D) cell culture system, cells are commonly cultivated by grafting cells onto a flat solid substrate in a culture medium, thereby allowing the cells to propagate as a monolayer after a few hours^22–25^. This 2D cell culture method enables easy, low-cost maintenance and manipulation of the conditions required for cell growth. However, the 2D cell culture approach does not fully duplicate the *in vivo* microenvironment of tissues, such as the growth microenvironment, cell signalling events, and other interactions with neighbouring cells or the extracellular matrix (ECM). When investigating new medical products, conventional 2D cell culture systems might provide misleading and non-predictive data, thereby leading to unexpected clinical trial results^26,27^. To overcome the disadvantages of the 2D cell culture platform, many recent studies have reported the use of three-dimensional (3D) cell culture systems. In previous studies, the cells cultured using a 3D culture system form aggregates or spheroids within a matrix, on a matrix, or in a suspension medium^27^.

Co-culture systems on a 3D platform mimic the tissue microenvironment of the *in vivo* model in terms of cell morphology and structural complexity, and also the biological processes and functions (e.g., proliferation, differentiation and gene or protein expression). Adipose cells in a 3D culture system enable co-culturing with a diverse array of cells, including macrophages, endothelial cells, and cancer cells^17,18,28,29^. Both physical and functional aspects of adipocytes that are co-cultured with the other cell types during differentiation and enlargement differed significantly in terms of cell morphology and cytokine expression compared to that of mono-cultured adipocytes^28^. In our previous study^30^, we also found that the expression levels of the metabolic pathway-related proteins from 3D co-cultured adipocytes with macrophages changed distinctively compared to 2D and 3D mono-cultured adipocytes. To clarify the metabolic differences between 2D and 3D mono/co-culture models, however, a comprehensive proteomic analysis of adipocyte proteome is required.

Recently, using liquid chromatography coupled with advanced tandem mass spectrometry (LC-MS/MS)^31–33^, numerous studies aiming to identify key effectors in the metabolic regulation of adipocytes have reported the results of quantitative proteomic analyses utilising diverse labelling techniques (e.g., isobaric tags for relative and absolute quantification [iTRAQ]^34–39^, tandem mass tags [TMT]^40,41^ and stable isotope labelling by amino acids in cell culture [SILAC]^42,43^). Here, we performed a global quantitative proteomic analysis of six 3T3-L1 cell types (preadipocytes, adipocytes, and co-cultured adipocytes with macrophages in 2D- and 3D-cell culture conditions) using iTRAQ-based 2D-nanoLC-ESI-MS/MS.

## Results & Discussion

### *In vitro* insulin resistance-induced 3D co-culture system

*In vitro* 2D mono-culture studies are considered to be a fundamental, accessible, effective, and promising means to identify the cellular mechanisms and key effectors related to metabolic syndromes. However, the 2D mono-culture approach fails to model the influence of the surrounding tissue architecture on adipocytes. Thus, attempts to develop *in vitro* 3D cell culture systems have been undertaken. In our previous study^30^, we demonstrated that the co-culture of adipocytes and macrophages in a 3D cell culture system results in changes in lipid and glucose metabolism, which is similar to that of the effect of GW9662 on insulin resistance in adipose tissue in diabetic mice. However, there is no clear reason why the insulin sensitivity in adipocytes induced by 3D co-culturing models with macrophages was shown to be consistent with the response observed *in vivo* in adipose tissue. In this study, we performed a quantitative proteomic analysis of six different 3T3-L1 cells (preadipocytes, adipocytes and each cell type co-cultured with macrophages in 2D or 3D cell culture conditions) under three different culture conditions (2D, 3D, and 3D co-culture of macrophages). This allowed us to quantitatively profile the adipocyte proteome, thereby identifying candidates that are directly or indirectly linked to the occurrence of insulin resistance.

**Figure 1:**
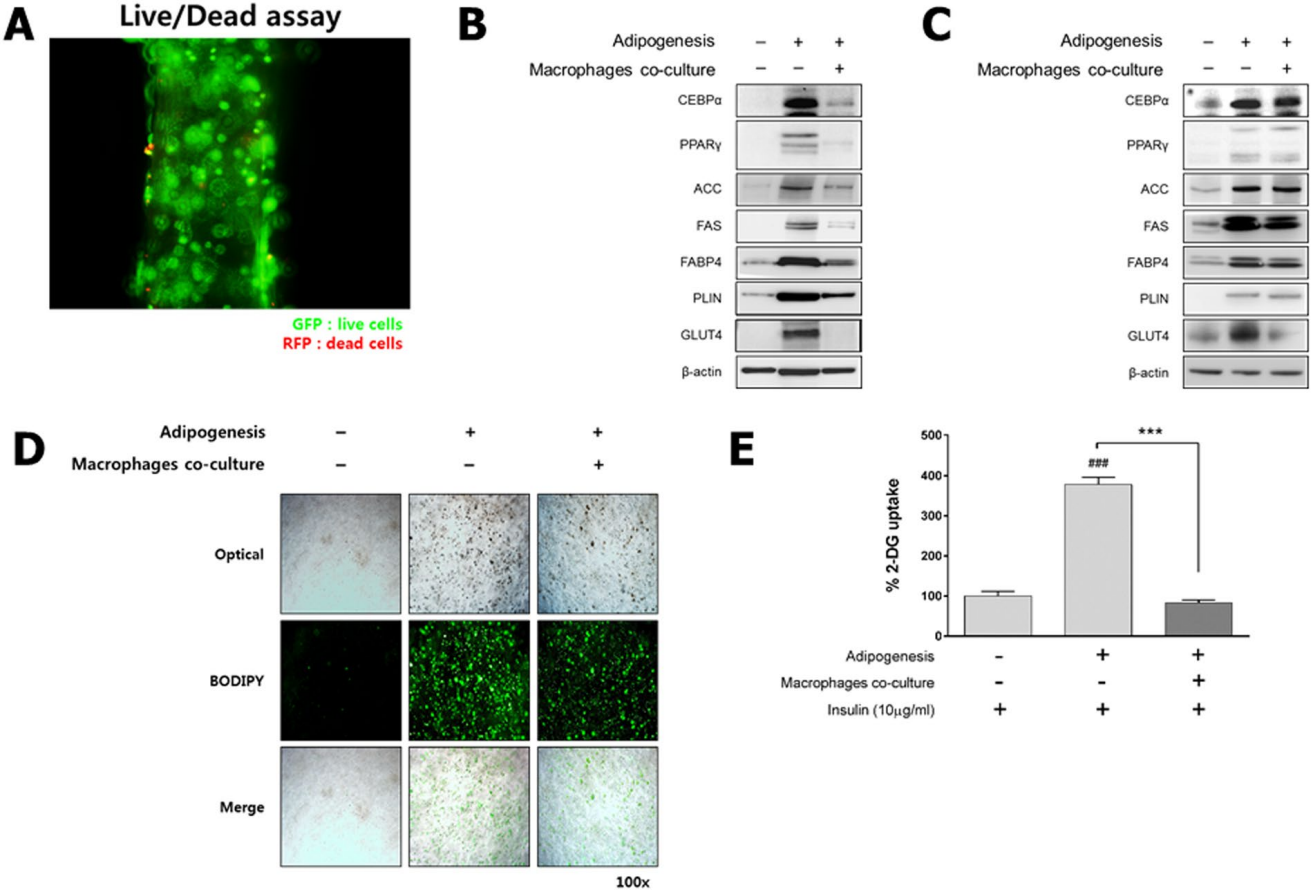
Fabrication of the insulin resistance-induced 3D co-culture model. For the 3D co-culture system, 3D scaffolds were fabricated using 3T3-L1 preadipocytes with or without 1% macrophages. (**A**) Fluorescent images of 3D mono-cultured 3T3-L1 preadipocytes at 7 days using a live/dead cell assay (magnification: 100×). Live cells stained green and dead cells stained red. (**B**) The protein concentrations of adipogenesis-related markers, such as CCAAT/enhancer binding protein alpha (C/EBPα), peroxisome proliferator-activated receptor gamma (PPARγ), acetyl-CoA carboxylase (ACC), fatty acid synthase (FAS), fatty acid binding protein 4 (FABP4), perilipin (PLIN), glucose transporter type 4 (GLUT4) and beta actin (β-actin), were assessed by Western blotting in the 2D and (**C**) 3D mono-/co-cultured model. (**D**) Fluorescent image of 3D mono-/co-cultured adipocytes by BODIPY lipid staining (magnification: 100×). (**E**) Level of glucose uptake in the 3D culture system. Results are expressed as the means ± S.E.M. (n = 4/group) for duplicate experiments. ^###^p < 0.001 vs insulin-treated 3D preadipocytes, ***p < 0.001 vs insulin-treated 3D adipocytes. Statistical significance was determined using the Student’s t-test or a one-way analysis of variance followed by Tukey’s multiple-comparison test; *p* < 0.05 was considered to be statistcally significant. Full-length blots are presented in Figs S1 and S2 in the Supporting Information.

We first prepared 3D-cultured 3T3-L1 preadipocytes that were fabricated in an alginate scaffold at a concentration of 1 × 10^6^ cells/mL. Based on a live/dead cell assay, preadipocytes in the 3D scaffold displayed good viability (Fig. 1A). A functional analysis of the 2D and 3D cell culture systems was performed by assessing the expression of adipogenesis-related markers (CEBPα, PPARγ, ACC, FAS, PLIN, FABP4 and GLUT4) during differentiation under mono-/co-culture conditions. Western blotting revealed that 2D mono-cultured preadipocytes were fully differentiated into adipocytes, but the differentiation of 2D co-cultured preadipocytes with macrophages in the trans-well plate was minimal (Fig. 1B). Interestingly, although the expression of proteins related to lipogenesis in both the 3D mono- and co-cultured cells culture was maintained, as in the 2D mono-cultured adipocytes, GLUT4 expression was markedly decreased (Fig. 1C). These findings are consistent with those of previous studies^44,45^. The distribution of lipid droplets in the 3D co-/mono-culture model as determined by BODIPY lipid staining indicated that 3T3-L1 preadipocytes were fully differentiated into adipocytes to an almost equivalent extent compared to the 3D mono-cultured adipocytes (Figs 1D and S4 in Supporting Information for 3D co-culture and 3D mono-culture model, respectively). In addition, to confirm the ratios of macrophages to the total number of cells, we performed a fluorescence-activated cell sorting (FACS) analysis. There were 243 macrophages in the 3D co-culture model (about 10% of the total number of cells), and there were an estimated 2,399 adipocytes (Fig. S5 in Supporting Information). These results of FACS-based cell counting are also consistent with that of BODIPY lipid staining (Figs S5–1 in Supporting Information). A glucose uptake assay demonstrated that glucose uptake in the 3D co-cultured adipocytes significantly decreased relative to that of the 3D mono-cultured adipocytes (Fig. 1E). These results are consistent with the observation that adipose tissue in diabetic mice increases insulin resistance because of macrophage infiltration, and also suggests that the 3D co-culture system has the potential to overcome the limitations of the 2D cell culture system, which merely differentiates cells into adipocytes^21,46^.

### Proteomic analysis of six different 3T3-L1 cells in 2D and 3D mono-/co-culture conditions using online 2D-nanoLC-ESI-MS/MS

**Figure 2:**
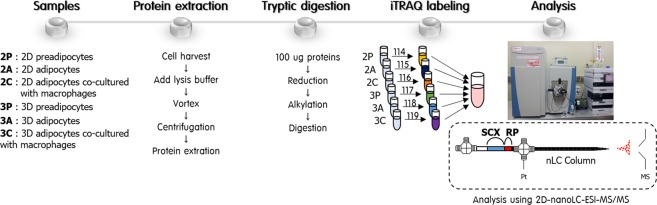
Experimental workflow for the comparative proteomic analysis of six different 3T3-L1 cells using 2D-nanoLC-ESI-MS/MS. Protein samples (100 µg) extracted from each cell were reduced, alkylated and digested with trypsin. Each tryptic digest was labelled with six-iTRAQ reagents after purification using SPE cartridges, and 2D-nanoLC-ESI-MS/MS analysis was performed to identify and quantify proteins.

**Figure 3:**
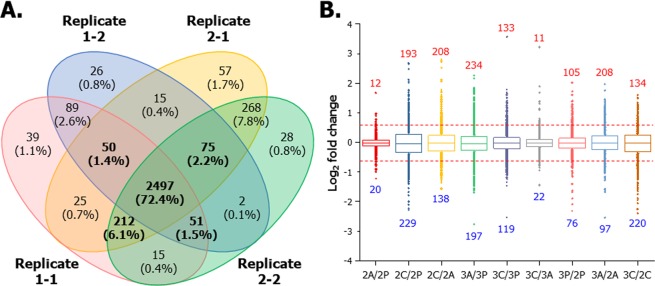
Data quality control. (**A**) Venn diagram showing the number of quantified proteins detected in two biological replicates from six different 2D and 3D mono-/co-cultured 3T3-L1 adipocytes with macrophages. The intersection shows a total of 2,885 proteins (83.6%) quantified in both biological replicates. (**B**) Diverse log_2_ iTRAQ ratios of six different 3T3-L1 cells were automatically normalized using Proteome Discoverer 1.4.1.14.

To profile the changes in the expression levels of adipocyte proteomes between 2D- and 3D-cultured 3T3-L1 models, we performed a global quantitative proteomic analysis of six different 3T3-L1 cells (2P, 2A, 2C, 3P, 3A and 3C) using iTRAQ-based online 2D-nanoLC-ESI-MS/MS and illustrated in Fig. 2. We included data quality controls in the proteomic analysis and determined the reproducibility of quantified protein groups from six different 3T3-L1 cells in four replicates (two technical and two biological replicates). As shown in Fig. 3A, 3,449 proteins from four replicates were successfully quantified with high confidence. Of these, 2,885 proteins (83.6%) were shared among four replicates with a minimum of three valid values (2,978 proteins from technical replicate 1 of biological replicate 1 [Replicate 1–1]; 2,805 proteins from technical replicate 2 of biological replicate 1 [Replicate 1–2]; 3,199 proteins from technical replicate 1 of biological replicate 2 [Replicate 2–1]; 3,148 proteins from technical replicate 1 of biological replicate 2 [Replicate 2–2]). The box plot shows the correlation of proteins among the six different 3T3-L1 cell lines in various ratios (Fig. 3B). The dotted red line indicates 1.5-fold increase or decrease in each ratio as the threshold value. Using the iTRAQ-based quantitative analysis, the levels of tens or hundreds of proteins expressed in 3T3-L1 cells changed because of differences in culture conditions. The quantified proteins are listed in the Supplementary Dataset (Supporting Table 1).

### Carbohydrate metabolism

**Figure 4:**
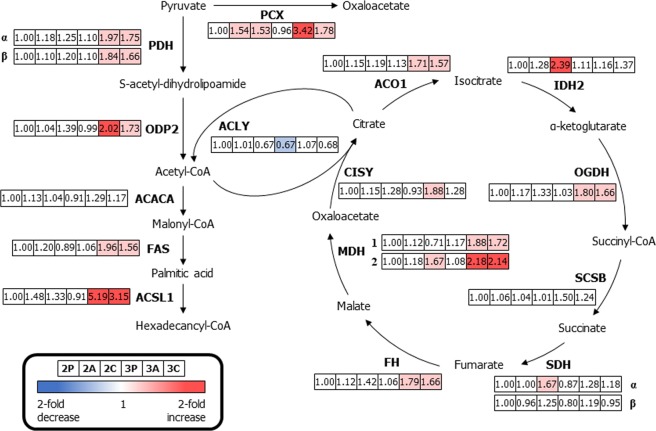
Differentially expressed proteins related to glycolysis and the citric acid cycle in six different 3T3-L1 cells. Down-regulated proteins expression compared to that of 2D mono-cultured preadipocytes (>1.5-fold change) are indicated in blue, and up-regulated proteins (>1.5-fold change) are indicated in pink. Boxes from the left to the right represent 2D mono-cultured preadipocytes (2P), 2D mono-cultured adipocytes (2A), 2D co-cultured adipocytes with macrophages (2C), 3D mono-cultured preadipocytes (3P), 3D mono-cultured adipocytes (3A) and 3D co-cultured adipocytes with macrophages (3C). [*Abbreviations: PDHα, pyruvate dehydrogenase E1 component subunit alpha; PDHβ, pyruvate dehydrogenase E1 component subunit beta; ODP2, ovule development protein 2; CISY, citrate synthase; ACO1, cytoplasmic aconitate hydratase; IDH2, isocitrate dehydrogenase; OGDH, 2-oxoglutarate dehydrogenase; SCSB, succinate-CoA synthetase subunit beta; SDHα, succinate dehydrogenase (ubiquinone) flavoprotein subunit; SDHβ, succinate dehydrogenase (ubiquinone) iron-sulphur subunit; FH, fumarate hydratase; MDH1, malate dehydrogenase, cytoplasmic; MDH2, malate dehydrogenase, mitochondria; PCX, pyruvate carboxylase; ACLY, ATP-citrate synthase; ACACA, acetyl-CoA carboxylase 1; FAS, fatty acid synthase; ACSL1, long-chain-fatty-acid-CoA ligase 1].

To understand how glucose homeostasis and adipocyte differentiation differ between the 2D and 3D cell-culture conditions, we confirmed the differential expression of six proteins in the 3T3-L1 cells that are involved in carbohydrate metabolism. The differential expression of 18 enzymes from six different 3T3-L1 cells (2P, 2A, 2C, 3P, 3A and 3C) were compared to those of 2P, and the results are summarised (Fig. 4). The expression levels of three proteins, acetyl-CoA carboxylase 1 (ACACA), succinate dehydrogenase iron-sulphur subunit (SDHβ), and succinate-CoA ligase subunit beta (SCSB), were not significantly different between the 2D and 3D cell culture systems. In the 2D cell culture system, the expression levels of four proteins (pyruvate carboxylase [PCX, 1.54- and 1.53-fold change for 2A and 2C], isocitrate dehydrogenase [IDH2, 2.39-fold change for 2C], malate dehydrogenase, mitochondrial [MDH2, 1.67-fold change for 2C], and succinate dehydrogenase flavoprotein subunit [SDHα, 1.67-fold change for 2C]) were up-regulated in 2D-cultured adipocytes in the presence or absence of macrophages. Conversely, most proteins expressed in the adipocytes that were 3D mono-/co-cultured with macrophages (except for the four proteins described above) were involved in carbohydrate metabolism (including PCX [3.42- and 1.78-fold change for 3A and 3C, respectively], pyruvate dehydrogenase E1 component subunit alpha [PDHα, 1.97- and 1.75-fold change for 3A and 3C, respectively], pyruvate dehydrogenase E1 component subunit beta [PDHβ, 1.84- and 1.66-fold change for 3A and 3C, respectively], the dihydrolipoyllysine-residue acetyltransferase component of pyruvate [ODP2, 2.02- and 1.73-fold change for 3A and 3C, respectively], FAS [1.96- and 1.56-fold change for 3A and 3C, respectively], long-chain-fatty-acid-CoA ligase 1 [ACSL1, 5.19- and 3.15-fold change for 3A and 3C, respectively], cytoplasmic aconitate hydratase [ACO1, 1.71- and 1.57-fold change for 3A and 3C, respectively], 2-oxoglutarate dehydrogenase [OGDH, 1.80- and 1.66-fold change for 3A and 3C, respectively], fumarate hydratase [FH, 1.79- and 1.66-fold change for 3A and 3C, respectively], malate dehydrogenase, cytoplasmic [MDH1, 1.88- and 1.72-fold change for 3A and 3C, respectively], MDH2 [2.18- and 2.14-fold change for 3A and 3C, respectively], and citrate synthase [CISY, 1.88-fold change for 3A]) and displayed at least a 1.5-fold change in expression levels. In particular, PCX plays a crucial role in the tricarboxylic acid (TCA) cycle. Pyruvate is introduced to the TCA cycle, and ATP citrate synthase (ACLY) allows acetyl-CoA derived from pyruvate to enter the TCA cycle. CISY catalyses the conversion of oxaloacetate into citrate. These enzymes are crucial for proper operation of the TCA cycle and they were significantly up-regulated, probably because of the production of citrate in the cytoplasm during differentiation and cell growth^10,35^. Furthermore, ACSL1 is required for both carbohydrate and fatty acid metabolism and was also up-regulated in 3A and 3C^39^. We demonstrated that the changes in expression levels of three proteins (CISY, FAS, and ACSL1) identified via iTRAQ-based quantitative assessments were similar to the results of Western blot analyses (Fig. S3A in Supporting Information).

### Fatty acid metabolism with β-oxidation

**Figure 5:**
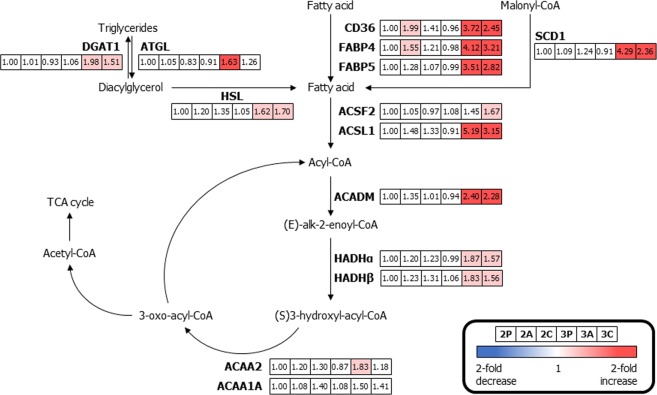
Differentially expressed proteins related to fatty acid metabolism involving β-oxidation in six different 3T3-L1 cells. Proteins down-regulated when compared to those of 2D mono-cultured preadipocytes (>1.5-fold change) are indicated in blue, and up-regulated proteins (>1.5-fold change) are indicated in pink. Boxes from the left to the right represent 2D mono-cultured preadipocytes (2P), 2D mono-cultured adipocytes (2A), 2D co-cultured adipocytes with macrophages (2C), 3D mono-cultured preadipocytes (3P), 3D mono-cultured adipocytes (3A) and 3D co-cultured adipocytes with macrophages (3C). (*Abbreviations: CD36, platelet glycoprotein 4; FABP4, fatty acid-binding protein, adipocyte; ACSF2, acyl-CoA synthetase family member 2, mitochondrial; ACSL1, long-chain-fatty-acid-CoA ligase 1; CPT1A, carnitine O-palmitoyltransferase 1, liver isoform; ACADM, medium-chain specific acyl-CoA dehydrogenase, mitochondrial; HADHα, trifunctional enzyme subunit alpha; HADHβ, trifunctional enzyme subunit beta; ACAA2, 3-ketoacyl-CoA thiolase; ACCA1A, 3-ketoacyl-CoA thiolase A; HSL, hormone-sensitive lipase; ATGL, patatin-like phospholipase domain-containing protein 2; DGAT1, diacylglycerol O-acyltransferase 1; SCD1, stearoyl-CoA desaturase 1).

Because lipids and their metabolites are implicated in regulating metabolic pathways leading to insulin resistance, the investigation of free fatty acids in adipocytes is important^3^. Fig. 5 shows a map of the key proteins involved in fatty acid metabolism and the expression dynamics of six different 3T3-L1 cells compared to the expression dynamics of 2D mono-cultured preadipocytes. Free fatty acids were transported across the plasma membrane and into the mitochondria using platelet glycoprotein 4 (CD36), FABP4, and fatty acid-binding protein 5 (FABP5)^7,35^. The expression levels of CD36 (1.99-, 3.72-, and 2.45-fold change for 2A, 3A, and 3C, respectively) and FABP4 (1.55-, 4.12-, and 3.21-fold change for 2A, 3A, and 3C, respectively) in 2D mono-cultured adipocytes and 3D-cultured adipocytes with/without macrophages were significantly up-regulated. The diverse enzymes involved in lipolysis and fatty acid metabolism, including FABP5 (3.51- and 2.82-fold change for 3A and 3C, respectively), diacylglycerol O-acyltransferase 1 (DGAT1, 1.98- and 1.51-fold change for 3A and 3C, respectively), patatin-like phospholipase domain-containing protein 2 (ATGL, 1.63-fold change for 3A), hormone-sensitive lipase (HSL, 1.62- and 1.70-fold change for 3A and 3C, respectively), stearoyl-CoA desaturase 1 (SCD1, 4.29- and 2.36-fold change for 3A and 3C, respectively), acyl-CoA synthetase family member 2, mitochondrial (ACSF2, 1.67-fold change for 3C), ACSL1 (5.19- and 3.15-fold change for 3A and 3C, respectively), mitochondrial medium-chain specific acyl-CoA dehydrogenase (ACADM, 2.40- and 2.28-fold change for 3A and 3C, respectively), trifunctional enzyme subunit alpha (HADHα, 1.87- and 1.57-fold change for 3A and 3C, respectively), trifunctional enzyme subunit beta (HADHβ, 1.83- and 1.56-fold change for 3A and 3C, respectively), and 3-ketoacyl-CoA thiolase (ACAA2, 1.83-fold change for 3A) varied considerably in 3D mono-cultured adipocytes and/or adipocyte 3D co-cultured with macrophages when compared to 2D mono-cultured preadipocytes. Enzymes that are activated in the fatty acid oxidation pathway (β-oxidation) were catalysed using acetyl-CoA from free fatty acids and they served to introduce acetyl-CoA into the TCA cycle. Proteins up-regulated during fatty acid metabolism might trigger excess acetyl-CoA production for the maintenance of intracellular homeostasis, which could affect mitochondrial proteins in the electron transport complexes, resulting in insulin sensitivity^47^. Western blotting subsequently confirmed these changes in expression of both FABP4 and ACADM, verifying the results using iTRAQ (Fig. S3B in Supporting Information).

### Other aspects of metabolism

The metabolism of adipose cells associated with insulin resistance, as well as glucose and fatty acid metabolism, are important for understanding obesity and metabolic syndromes. Furthermore, the adipokines or cytokines induced during adipogenesis and the operation of the mitochondrial electron transport chain (ETC) are also key contributors to obesity^10,23,39^. To assess protein expression dynamics among the six different 3T3-L1 cells, we quantified the expression levels of the enzymes in relation to adipogenesis and the ETC pathways. As shown in Fig. S6 in the Supporting Information, four proteins related to the adipogenesis pathway were quantified in six different 3T3-L1 cells. The measured perilipin-1 (PLIN1, 2.44- and 2.21-fold change for 3A and 3C, respectively), lipoprotein lipase (LPL, 2.80- and 2.06-fold change for 3A and 3C, respectively), transmembrane protein 120A (TMEM120A, 3.00- and 1.93-fold change for 3A and 3C, respectively), and acyl-CoA-binding protein (ACBP, 1.83- and 1.60-fold change for 3A and 3C, respectively) were significantly over-expressed in 3D mono-cultured adipocytes and adipocytes 3D co-cultured with macrophages. Among these differentially expressed proteins, PLIN1 and LPL correlate with insulin sensitivity^48,49^ and TMEM120A are required for adipocyte differentiation^50^.

To determine the influence of mitochondria and ROS, we analysed the expression dynamics of mitochondrial proteins involved in the ETC and ATP synthesis. Four proteins involved in the function of ETC complexes were examined in six different 3T3-L1 cells and compared to the expression levels in 2D mono-cultured preadipocytes (Fig. S7 in the Supporting Information). Electron transfer flavoprotein subunit alpha (ETFA, 1.80- and 1.80-fold change for 3A and 3C, respectively), which transfers electrons from NADH to CoQ across the mitochondrial membrane in ETC complex I^10,51^, was significantly up-regulated (greater than 1.5-fold) in adipocytes that were 3D mono- or co-cultured with macrophages. Additionally, cytochrome c (CYC, 1.80-fold change for 3A) and ATP synthase mitochondrial F1 complex assembly factor 2 (ATPF2, 1.86- and 1.64-fold change for 3A and 3C, respectively) were significantly up-regulated in 3D-cultured adipocytes with/without macrophages, which was similar to the dynamics that were observed for other metabolic pathways. The expression of ADP/ATP translocase 2 (ADT2, 1.77- and 1.96-fold change for 2C and 3A, respectively) was increased not only in 3D mono-cultured adipocytes, but also in 2D co-cultured adipocytes with macrophages. High expression of the enzymes involved in the ETC and ATP synthase might lead to mitochondrial dysfunction or metabolic disorders in 3D cell culture conditions, which is similar to observations in the *in vivo* system^10,52^.

### Correlational network analysis of up- and down-regulated proteins in 3D co-cultured adipocytes with macrophages versus other cell models

**Figure 6:**
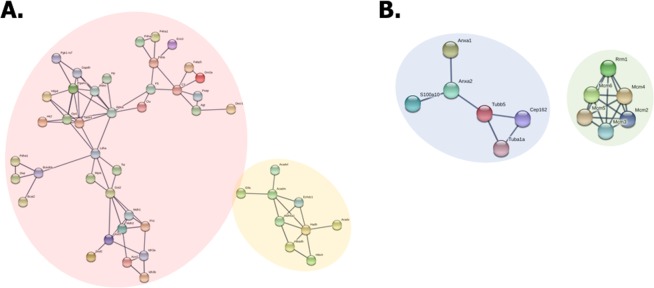
The correlated network analysis of up-regulated and down-regulated proteins in adipocytes 3D co-cultured with macrophages (3C) versus 2D mono-cultured preadipocytes (2P). Up-regulated and down-regulated proteins were determined based upon an observed >1.5-fold change in expression among biological and technical replicates. The functional interactions of proteins were predicted using the STRING network analysis program. (**A**) The network of up-regulated proteins in the pink circle is associated with glycolysis/gluconeogenesis and the TCA cycle, and the yellow circle consists of proteins involved in fatty acid metabolism. (**B**) The network of down-regulated proteins in the blue circle is associated with cellular component organization, and the proteins in the green circle are involved in DNA replication and regulation of the cell cycle.

**Figure 7:**
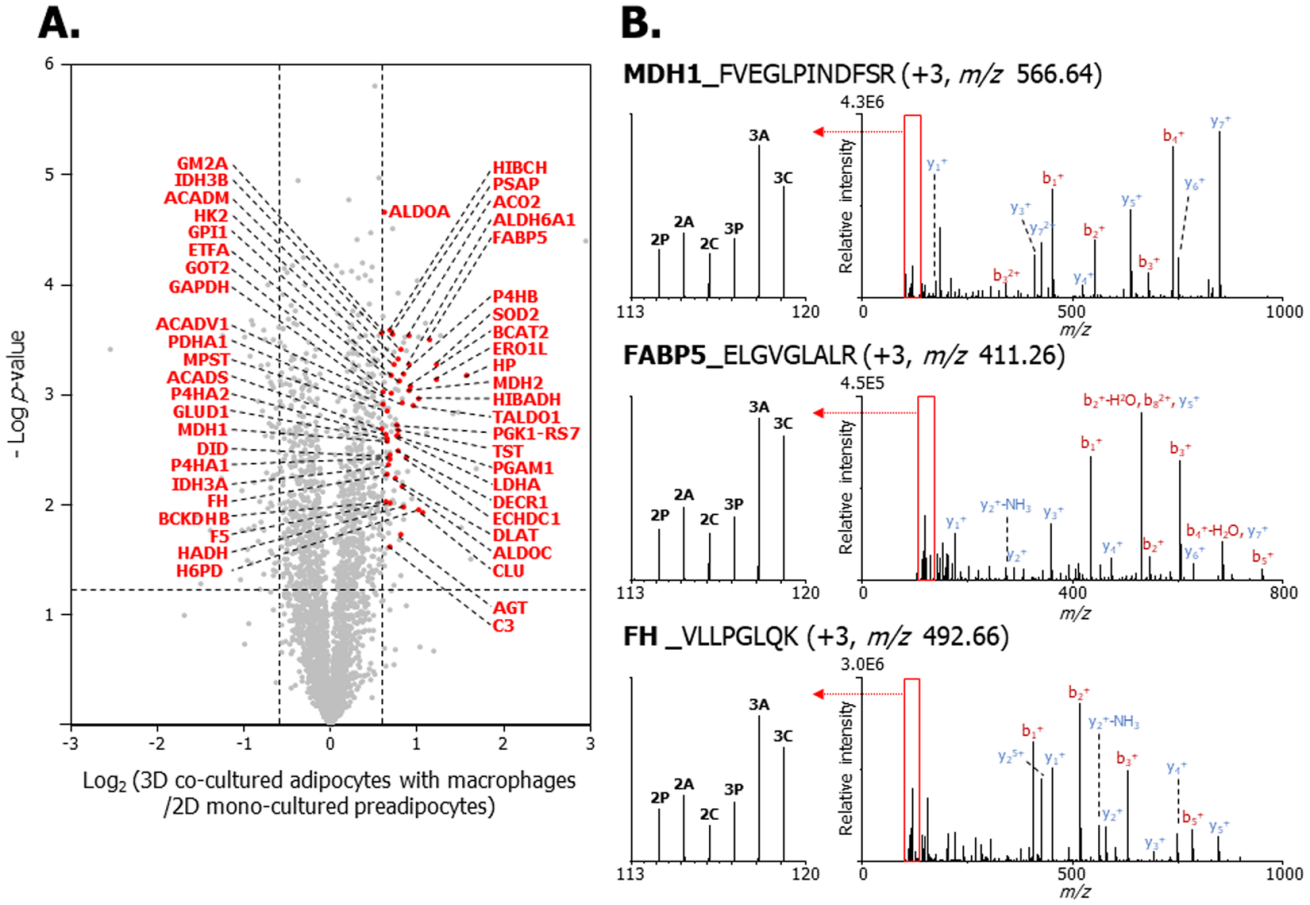
Volcano plot and MS/MS spectra of up-regulated proteins expressed in 3D co-cultured adipocytes with macrophages (3C) versus 2D mono-cultured preadipocytes (2P). (**A**) Volcano plot of quantified proteins between 3C and 2P illustrates the log_2_ 3C/2P iTRAQ ratio (x-axis) and –log p-value (y-axis). The non-axial vertical lines indicate ±1.5-fold change, and the non-axial horizontal line indicates *p = *0.05, which is the statistical threshold for measuring differentially expressed proteins. The 48 proteins denoted in red among 124 up-regulated proteins were included in the cluster. (**B**) MS/MS spectra of MDH1 (FVEGLPINDFSR), FABP5 (ELGVGLALR) and FH (VLLPGLQK), which are involved in glycolysis/gluconeogenesis, TCA cycle, and fatty acid metabolism. Left spectra indicate the iTRAQ reporter ions. 2P, 114.11; 2D mono-cultured adipocyte (2A), 115.11; 2D co-cultured adipocytes with macrophages (2C), 116.11; 3D mono-cultured preadipocytes (3P), 117.11; 3D mono-cultured adipocyte (3A), 118.11; 3C, 119.11.

To confirm the protein–protein interactions that occur in adipocytes that are in 3D co-culture with macrophages, the quantified proteins were analysed using STRING. Briefly, 48 up-regulated and 12 down-regulated proteins (which were among the top 124 up-regulated and 66 down-regulated proteins) were clustered (Tables S1 and S2 in the Supporting Information). As shown in Fig. 6A, the cluster indicated by the pink circle correlates with glycolysis, gluconeogenesis, and the TCA cycle. The components (including PDHα, MDH1/2, and FH) contribute to carbohydrate metabolism, which supports the results shown in Fig. 4. The other cluster indicated by the yellow circle includes nine proteins that are involved in mitochondrial fatty acid metabolism, including ETFA, very long-chain specific acyl-CoA dehydrogenase (VLCAD), ACADM, ethylmalonyl-CoA decarboxylase (ECHDC1), methylmalonate-semialdehyde dehydrogenase (ALDH6A1), hydroxyacyl-coenzyme A dehydrogenase (HADH), 3-hydroxyisobutyrate dehydrogenase (HIBADH), short-chain specific acyl-CoA dehydrogenase (SCAD), and 3-hydroxyisobutyryl-CoA hydrolase (HIBCH). These up-regulated proteins are involved in fatty acid beta-oxidation in the mitochondria^53^, and correlate with the proteomic data, as shown in Fig. 5. In Fig. 7, 48 up-regulated proteins are indicated by red dots. The abbreviations of proteins in a volcano plot (Fig. 7A), along with MS/MS spectra of MDH1 (FVEGLPINDFSR, +3, *m/z* 566.64), FABP5 (ELGVGLALR, +3, *m/z* 411.26), and FH (VLLPGLQK, +3, *m/z* 492.66) are described (Fig. 7B). Two clusters were formed by 12 down-regulated proteins in adipocytes that were 3D co-cultured with macrophages (Fig. 6B). Six down-regulated proteins denoted by the blue circle are involved in cellular component organisation. Among these proteins, ANXA1 and ANXA2 are involved in glucose and lipid metabolism and are implicated in insulin sensitivity^54^. Moreover, the development of insulin resistance in ANXA1-deficient mice has been reported in a previous study^55^. The cluster denoted by the green circle consisting of MCM family proteins (MCM2, 3, 4, 5, 6) are required to initiate eukaryotic genome replication^56^ and are involved in DNA replication and the cell cycle. The abovementioned down-regulated proteins are denoted by blue dots and the abbreviations of the proteins in the volcano plot (Fig. S8A in the Supporting Information). Selected MS/MS spectra of MCM2 (DNNDLLLFILK, +3, *m/z* 642.72), ANXA1 (FLENQEQEYVQAVK, +2, *m/z* 1167.13), and TUBB5 (ISVYYNEATGGK, +3, *m/z* 637.35) are described in Fig. S8B in the Supporting Information. These results show that the interactions among down-regulated proteins are associated with a decrease in cell proliferation and the development of insulin resistance in the 3D co-culture system of adipocytes and macrophages.

### Functional annotation of differentially expressed proteins in 3D co-cultured adipocytes with macrophages versus 2D mono-cultured adipocytes

**Figure 8:**
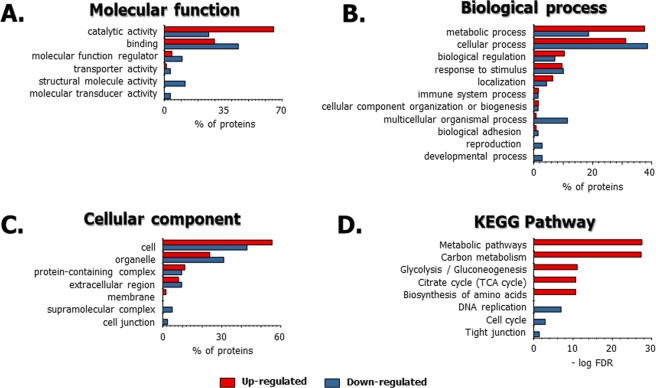
Functional annotations of up-regulated and down-regulated proteins in 3D co-cultured adipocytes with macrophages compared to those in the 2D mono-cultured adipocytes using the PANTHER classification system and STRING. (**A**) Molecular function, (**B**) biological process, (**C**) cellular component, (**D**) KEGG pathway. For the classification of quantified proteins, 124 up-regulated and 67 down-regulated proteins from a total of 2,885 quantified proteins were used. The red and blue rods show the percentage (%) and –log FDR value of up-regulated and down-regulated proteins, respectively. The KEGG pathways for the up-regulated and down-regulated proteins are listed in Supporting Table 2.

To determine the effects of the 3D adipocyte culturing technique on the physiology of the adipocyte proteome, we performed a bioinformatic-based functional analysis of the differentially expressed proteins from adipocytes that were 3D co-cultured with macrophages versus the typical cell culture system (2D mono-cultured adipocytes). Among the proteins analysed, 124 and 67 proteins were up- and down-regulated, respectively, in 3D-cultured adipocytes with macrophages compared to the expression levels in 2D mono-cultured adipocytes. The molecular functions of those up-/down-regulated proteins were implicated in the catalytic activity, binding, and other activities related to structural molecule, transporter, and molecular transducer (Fig. 8A). Furthermore, many up-regulated and down-regulated proteins were found to be involved in a metabolic process, cellular process, and biological regulation (Fig. 8B). The major cellular components were assigned to the parts of the cell, organelle, and protein-containing complex region (Fig. 8C). The major functions of the up-regulated and down-regulated proteins in adipocytes that are 3D co-cultured with macrophages relate to the metabolism of glucose and fatty acids in adipose cells during differentiation^34,35^. Based on a KEGG pathway analysis of up-regulated and down-regulated proteins (Fig. 8D), the up-regulated pathways in adipocytes that were 3D co-cultured with macrophages relate to metabolism during adipocyte differentiation. This includes such metabolic pathways as carbon metabolism, glycolysis/gluconeogenesis, the TCA cycle, and biosynthesis of amino acids. These results are similar to those of previous studies^57,58^. Conversely, the down-regulated pathways in adipocytes 3D co-cultured with macrophages were associated with cell proliferation, such as DNA replication, cell cycle regulation and tight junctions. The KEGG pathways for the up-regulated and down-regulated proteins are listed in Supporting Table 2. Based on these results, it is possible that adipocytes 3D co-cultured with macrophages can provide a suitable *in vitro* obesity model.

### Functional annotation of 3D versus 2D co-cultured adipocytes with macrophages

To understand the diverse functions of the up-regulated and down-regulated proteins in adipocytes that were 3D co-cultured with macrophages compared to adipocytes that were 2D co-cultured with macrophages, we submitted 134 up-regulated and 220 down-regulated proteins to the online tool PANTHER classification system and STRING. With regard to molecular function, many up-regulated and down-regulated proteins in adipocytes 3D co-cultured with macrophages are involved in catalytic activity, binding, structural molecular activity, and molecular function regulation (Fig. S9A in the Supporting Information). A biological process analysis revealed that up-regulated and down-regulated proteins in adipocytes 3D co-cultured with macrophages included metabolic processes, cellular processes, and localisation (Fig. S9B in the Supporting Information). Cellular components were related to the parts of the cell, organelle, and protein-containing complex region (Fig. S9C in the Supporting Information). The KEGG pathways for the up-regulated proteins in adipocytes 3D-cultured with macrophages were different than those of the 2D co-cultured adipocytes (Fig. S9D in the Supporting Information). The KEGG pathways that were enriched for the up-regulated proteins included carbon metabolism, metabolic pathways, propanoate metabolism, PPAR signalling pathways, and biosynthesis of amino acids, while down-regulated proteins in adipocytes 3D co-cultured with macrophages were mainly involved in oxidative phosphorylation and thermogenesis. From these results, we propose that the adipocytes that were 3D co-cultured with macrophages represent a useful *in vitro* obesity model.

### Functional annotation of adipocytes 3D co-cultured with macrophages versus 3D mono-cultured adipocytes

To understand the changes in the microenvironment when adipocytes are co-cultured with macrophages, we performed a functional annotation analysis by comparing adipocytes 3D co-cultured with macrophages to 3D mono-cultured adipocytes. Among the 2,885 proteins analysed, 11 up-regulated and 22 down-regulated proteins in adipocytes 3D co-cultured with macrophages were identified based upon a comparison to the expression profile of 3D mono-cultured adipocytes using the PANTHER classification system and STRING. The molecular functions of the differentially expressed proteins mainly related to binding, catalytic activity, and transporter activity (Fig. S10A in the Supporting Information). For the biological processes, the majority of differentially expressed proteins were involved in cellular and metabolic processes (Fig. S10B in the Supporting Information), and the localisation of the proteins was predicted to be in the cell, extracellular region, and organelles (Fig. S10C in the Supporting Information). These functional annotations supported our previous results that were obtained for adipocytes that were 3D versus 2D co-cultured with macrophages or for adipocytes that were 3D co-cultured with macrophages versus 2D mono-cultured adipocytes. Interestingly, fatty acid metabolism, biosynthesis of unsaturated fatty acids and other functions in the KEGG pathway were down-regulated in adipocytes 3D co-cultured with macrophages (Fig. S10D in the Supporting Information and Supporting Table 2). These findings suggest that macrophage infiltration might be affected by metabolic processes and insulin resistance and that the enzymes involved in fatty acid metabolism are downregulated. We suggest that the co-culture of adipocytes and macrophages during differentiation and cell growth reduces lipolysis of adipocytes by altering cytokine production in macrophages.

## Conclusions

This is the first report of a comprehensive quantitative proteomic analysis of a 3D *in vitro* co-culture model of 3T3-L1 cells and RAW264.7 macrophages. This study has focused primarily on metabolic mechanisms. We performed a high-throughput proteomic analysis of six different 3T3-L1 cells and explored differential protein expression between the 2D and 3D co-cultured adipocyte platform, in addition to preforming a functional annotation. Our analysis identified differentially expressed proteins that are involved in glucose metabolism, fatty acid oxidation, adipogenesis, and the ETC pathway. In particular, the expression levels of proteins involved in diverse metabolic processes were up-regulated in adipocytes 3D co-cultured with macrophages compared to the levels in the 2D-cultured adipocytes. Furthermore, the activation of major cellular metabolism pathways leads to insulin resistance caused by the adipocyte hypertrophy, energy accumulation, and mitochondrial dysfunction. The 3D *in vitro* co-culture model consisting of 3T3-L1 cells and RAW 264.7 macrophages along with comprehensive proteomic datasets could widen the potential applicability of mechanical studies and drug screening associated with insulin resistance. To better understand the effects of 3D cell culturing on the adipocyte proteome, however, the development of a fluorescence-activated cell sorting (FACS)-based method for isolating adipocyte cells from macrophages in 3D co-culture systems is required for future study, and an in-depth quantitative proteomic analysis may provide insights into the biological alterations of the adipocyte proteome in 3D cell culture systems.

## Materials and Methods

### Materials and chemicals

Critical materials and chemicals are listed in Method S1, Supporting Information.

### Cell culture and differentiation in the 2D system

3T3-L1 cells (ATCC CL-173, mouse adipocyte) were seeded at 1 × 10^5^ cells/well (under plate), and RAW264.7 macrophages (ATCC, TBI-71) were seeded at 1 × 10^4^ cells/well (upper plate) in a trans/6-well plate. Cell densities were determined using a disposable haemocytometer-based cell counter (SKC Co. Ltd., Seoul, Korea) with the aid of an inverted microscope (Eclipse TE2000-U, Nikon, Tokyo, Japan). 3T3-L1 cells and RAW264.7 macrophages were maintained in Dulbecco’s modified Eagle’s medium (DMEM) supplemented with 10% fetal bovine serum (FBS), 100 μg/mL penicillin and 100 μg/mL streptomycin at 37 °C under 5% CO_2_. The 3T3-L1 cells with and without RAW264.7 macrophages were differentiated into mature adipocytes in the same medium containing 20 μg/mL insulin, 0.5 mM isobutylmethylxanthine and 1 μM dexamethasone for 3 days. The medium was then replaced with a medium containing 20 μg/mL insulin for 2 days, and the cells were cultured for 1 day in the culture medium.

### 3D scaffold fabrication using the cell-plotting system

In our previous study, we developed a 3D cell printing system to assemble 3D-dispensing structures^30^. Briefly, this system consists of an x-y-z stage, a dispenser, a syringe nozzle, a compression controller and a computer system. The dispenser is the tank reservoir used to hold the cell-hydrogel mixture and it has a 10 × 10 × 10 cm space to fabricate the 3D architecture. The computer system controls the pressure, feeding speed, strand size and shape of the scaffold. The pressure and speed of the cell-plotting system were controlled at 650 kPa and 150 mm/s, respectively. Constant air pressure was applied to the dispenser, and a 3D scaffold was plotted layer-by-layer on the x-y-z stage. The scaffold pattern was designed with an orthogonal orientation. Alginate mixed with collagen, gelatin and cells was placed in a dispenser with a 100–400-μm nozzle for cell printing. The resulting alginate constructs were composed of 10 layers (2–4 mm) in a 3 × 3 cm square. The scaffold fabrication equipment 3D Bio Mohno Master (M4T, Daegu, Republic of Korea) and related software were used at the Korea Research Institute of Chemical Technology (KRICT).

### Cell culture and differentiation in the 3D culture system

The seeding densities (1 × 10^6^ cells/mL) of 3T3-L1 preadipocytes and RAW264.7 macrophages (1 × 10^4^ cells/mL) were adjusted using a disposable haemocytometer-based cell counter (SKC Co. Ltd., Seoul, Korea) with the aid of an inverted microscope (Eclipse TE2000-U, Nikon, Tokyo, Japan). Cells/hydrogel mixtures were plotted using the cell plotting system. The cells in the plotted hydrogel were observed by a live/dead cell assay kit (Invitrogen, Carlsbad, CA) using fluorescence microscopy (Eclipse TE2000-U, Nikon, Tokyo, Japan). Fabricated scaffolds were differentiated using the differentiation medium containing 20 μg/mL insulin, 0.5 mM isobutylmethylxanthine and 1 μM dexamethasone for 3 days, which was then replaced with a medium containing 20 μg/mL insulin for 2 days. Cells were fully differentiated, and the medium was exchanged with an insulin-free medium containing 10% FBS.

### Lipid droplet fluorescent staining

To confirm the differential rates of adipocytes from preadipocytes on 3D mono-/co-culture system, we performed a Lipid droplet fluorescent staining and methodological details are provided in Method S2, Supporting Information.

### Fluorescence-activated cell sorting (FACS) analysis

We performed a FACS analysis to measure the ratios of macrophages to the total number of cells in 3D co-culture model and detailed in Method S3, Supporting Information.

### Western blotting

The cell lysates from the 2D cultured system and 3D scaffolds were treated in a PRO-PREP protein extraction solution (iNtRON Biotechnology Inc., Seoul, Korea). Protein samples were loaded onto 4–12% NuPage Bis-Tris Mini Gels (Invitrogen, Carlsbad, CA) and transferred to a polyvinylidene difluoride membrane (Amersham Biosciences, Piscataway, NJ). The blocked membrane was then incubated with primary antibodies against CCAAT/enhancer binding protein α (CEBPα), peroxisome proliferator-activated receptor gamma (PPARγ), acetyl-CoA carboxylase (ACC), fatty acid synthase (FAS), perilipin (PLIN), fatty acid binding protein (FABP4), glucose transporter 4 (GLUT4), acyl-CoA synthetase long chain family member 1 (ASCL1), citrate synthase (CISY), β-actin (Cell Signaling, Danvers, MA) and acyl-CoA dehydrogenase medium chain (ACADM, GeneTex. Irvine, CA). Immunoreactive bands were observed using a chemiluminescent reagent from the Supersignal West Dura Extended Duration Substrate Kit (Amersham Biosciences, Piscataway, NJ). Protein bands were visualized using chemiluminescence (BIORAD Inc., Seoul, Korea and UVITEC Ltd., Cambridge, UK).

### Sample preparation for proteomic analysis

Each 3T3-L1 in 2D/3D cell culture system was collected into individual tubes and lysed by adding 500 μL of lysis buffer composed of 50 mM HEPES, 70 mM potassium acetate, 5 mM magnesium acetate, one tablet of protease inhibitor cocktail and 0.2% n-dodecyl-beta-D-maltoside in water. For protein extraction from six of 2D- and 3D-cultured 3T3-L1 cells, each mixture of cells and lysis buffer were vortexed for 1 min and then incubated on ice for 4 min. This process was repeated twice. The remaining unbroken cells and debris were removed by centrifugation at 4 °C and 12,000 × *g* for 15 min. The protein concentration of the cell lysates was quantified using the Bradford assay with Coomassie protein assay reagent. A standard curve was made using bovine serum albumin (BSA) as a control.

### Protein digestion and iTRAQ labelling

One-hundred micrograms of extracted proteins from the 2D- and 3D-cultured 3T3-L1 cells (six types) were transferred into a new tube. The six different cell lysates were reduced utilizing 50 mM ammonium bicarbonate (ABC) solution with 10 mM DL-dithiothreitol (DTT) for 2 h at 37 °C and alkylated by adding iodoacetamide (IAA) to a final concentration of 20 mM for 30 min at room temperature (RT) in the dark. To remove the remaining IAA in solution, L-cysteine was added at a final concentration of 40 mM for 30 min at RT. Protein samples were proteolytically digested with trypsin at a ratio of 1:20 (enzyme:protein) at 37 °C for 18 h. Each peptide solution was purified using HLB cartridges (1 cc, 10 mg) and then dried using a concentrator (Eppendorf, Concentrator plus) under a vacuum. Each of the dried samples were reconstituted with 30 μL of 0.5 M triethylammonium bicarbonate (TEAB) and 50 μL of isopropanol-suspended iTRAQ reagent containing the reporter ion from 114 to 119 (8-plex iTRAQ reagents). Six samples were labelled with the respective tags as follows: 2D mono-cultured preadipocytes (2P)-114 reagent, 2D mono-cultured adipocytes (2A)-115 reagent, 2D co-cultured adipocytes with macrophage (2C)-116 reagent, 3D mono-cultured preadipocytes (3P)-117 reagent, 3D mono-cultured adipocytes (3A)-118 reagent and 3D co-cultured adipocytes with macrophage (3C)-119 reagent. All samples were incubated for 2 h at RT before adding an equal amount of each sample into a new tube and drying using a concentrator under a vacuum. The iTRAQ-labelled samples reconstituted with 1 mL of water were purified using an HLB cartridge (1 cc, 30 mg) and dried using a concentrator under a vacuum. The iTRAQ-labelled peptides were reconstituted with 0.1% formic acid (FA) solution and analysed using 2D-nLC-ESI-MS/MS.

### Online 2D-nanoLC-ESI-MS/MS Analysis

We analysed the resulting digests using online 2D-nanoLC-ESI-MS/MS^59,60^, which is shown in Fig. 2. Additional methodological details are provided in Method S4, Supporting Information.

### Data analysis

iTRAQ-labelled peptides were identified and quantified by comparing MS/MS spectra against the *Mus musculus* database (mouse, UP000000589, updated on 01/18/2019) from UniProt (http://www.uniprot.org/) with a false discovery rate (FDR) of 0.01 using Proteome Discoverer 1.4.1.14 (Thermo Scientific, Bremen, Germany). To perform a data dependence analysis, the MS parameters were set to 20 ppm of a precursor peptide ion mass tolerance and 0.02 Da of a fragment peptide ion mass tolerance. Two missed cleavages were allowed. iTRAQ-8plex labelling of the N-terminus and carbamidomethylation of cysteine residues were set as the fixed modifications. The iTRAQ-8plex labelling of lysine residues, oxidation of methionine and phosphorylation of serine, threonine and tyrosine were all set as variable modifications. The quantified proteins obtained from six different 3T3-L1 cells were normalized against 20 median proteins using an experimental bias of the quantification method and were identified with at least two unique peptides per protein in Proteome Discoverer 1.4.1.14. Data processing and statistical analysis were carried out using Perseus 1.5.8.5. The iTRAQ ratios of proteins were transformed by log_2_(x) and filtered by a minimum of three valid values. The missing values in ratios of reporter ions corresponding to each 3T3-L1 cell cultured in the 2D/3D system were imputed using a normal distribution. Subsequently, a one-sample t-test was carried out to obtain the statistical significance among biological and technical replicates. The significance threshold used was a ±1.5-fold change and *p* < 0.05. The resulting data were exported to Microsoft Excel, and a Venn diagram was generated using the online tool Venny 2.1 (http://bioinfogp.cnb.csic.es/tools/venny/index.html). Protein classification was performed using the PANTHER classification system (http://www.pantherdb.org/tools/) based on functional annotations for cellular component, biological process, protein class and molecular function. The search tool for protein-protein interactions and the Kyoto Encyclopedia of Genes and Genomes (KEGG) pathway analysis were used to identify interacting genes and proteins (STRING, https://string-db.org/).

## Supplementary information

Supporting Information

Supporting Table 1

Supporting Table 2

## Data Availability

The datasets generated during the current study are available in PRIDE, accession number: PXD014437. https://www.ebi.ac.uk/pride/archive/login.
