# Supplementary material for: iTRAQ-Based Quantitative Proteomic Comparison of 2D and 3D Adipocyte Cell Models Co-cultured with Macrophages Using Online 2D-nanoLC-ESI-MS/MS
Source: Sci Rep. 2019 Nov 14;9:16746. doi: 10.1038/s41598-019-53196-0 (PMC6856061; doi:10.1038/s41598-019-53196-0)
Supplement: Supplementary file 1 — Supporting Information [file 41598_2019_53196_MOESM1_ESM.docx]

**Supporting Information**

iTRAQ-Based Quantitative Proteomic Comparison of 2D and 3D Adipocyte Cell Models Co-cultured with Macrophages Using Online 2D-nanoLC-ESI-MS/MS

Sun Young Lee,^1,§^ Sung Bum Park,^2,§^ Young Eun Kim,^2^ Hee Min Yoo,^3^ Jongki Hong,^1^ Kyoung-Jin Choi,^2^ Ki Young Kim,^2,*^ Dukjin Kang^3,*^

^1^College of Pharmacy, Kyung Hee University, Seoul, 02447, Republic of Korea

^2^Therapeutics & Biotechnology Division, Korea Research Institute of Chemical Technology, 141 Gajeong-ro, Yuseong-gu, Daejeon 305-600, Republic of Korea

^3^Center for Bioanalysis, Division of Chemical and Medical metrology, Korea Research Institute of Standards and Science, Daejeon, 34113, Republic of Korea

^§^Both authors contributed equally to this study

**Supporting Figure**

**Figure S1.** Full-length blots corresponding to Fig.1B in the main text.

**Figure S2.** Full-length blots corresponding to Fig.1C in the main text.

**Figure S3.** Validation of differentially expressed proteins associated with carbohydrate and fatty acid metabolisms in 2D and 3D culture system using western blot analysis.

**Figure S3-1.** Full-length blots corresponding to Fig.S3 in Supporting Information.

**Figure S4.** Lipid droplet fluorescent images of the 3D mono-cultured adipocytes differentiated from 3D mono-cultured preadipocytes

**Figure S5.** FACS analysis of 3T3-L1 adipocytes and red dye stained Macrophages were co-cultured for 8 days.

**Figure S5-1.** Lipid droplet fluorescent images of the adipocytes 3D co-cultured with macrophages.

**Figure S6.** Expression levels of iTRAQ-labeled proteins associated to adipogenesis from six different 3T3-L1 cells cultured in the 2D and 3D systems.

**Figure S7.** Expression levels of four different iTRAQ-labeled proteins related to electron transport chain and ATP synthase from six different 3T3-L1 cells cultured in the 2D and 3D systems.

**Figure S8.** Volcano plot and MS/MS spectra of down-regulated proteins in 3D co-cultured adipocytes with macrophages versus 2D mono-cultured preadipocytes.

**Figure S9.** Functional annotation of up-regulated and down-regulated proteins in 3D co-cultured adipocytes with macrophages compared to those in 2D ones using the PANTHER classification system and STRING.

**Figure S10.** Functional annotation of up-regulated and down-regulated proteins in 3D co-cultured adipocytes with macrophages compared to those in 3D mono-cultured adipocytes using the PANTHER classification system and STRING.

**Supporting Tables**

Along with Table S1 and S2, a total of proteins identified and quantified are shown in a supporting Excel file.

**Table S1.** Lists of up-regulated proteins included in the network analysis in 3D co-cultured adipocytes with macrophages.

**Table S2.** Lists of down-regulated proteins included in the network analysis in 3D co-cultured adipocytes with macrophages.

**Supporting Method**

**Method S1.** Materials and chemicals

**Method S2.** Lipid droplet fluorescent staining

**Method S3.** Fluorescence-activated cell sorting (FACS) analysis

**Method S4.** Online 2D-nanoLC-ESI-MS/MS for shotgun analysis of 2D- and 3D-mono/co-cultured adipocyte proteome

**Supporting Figure S1**


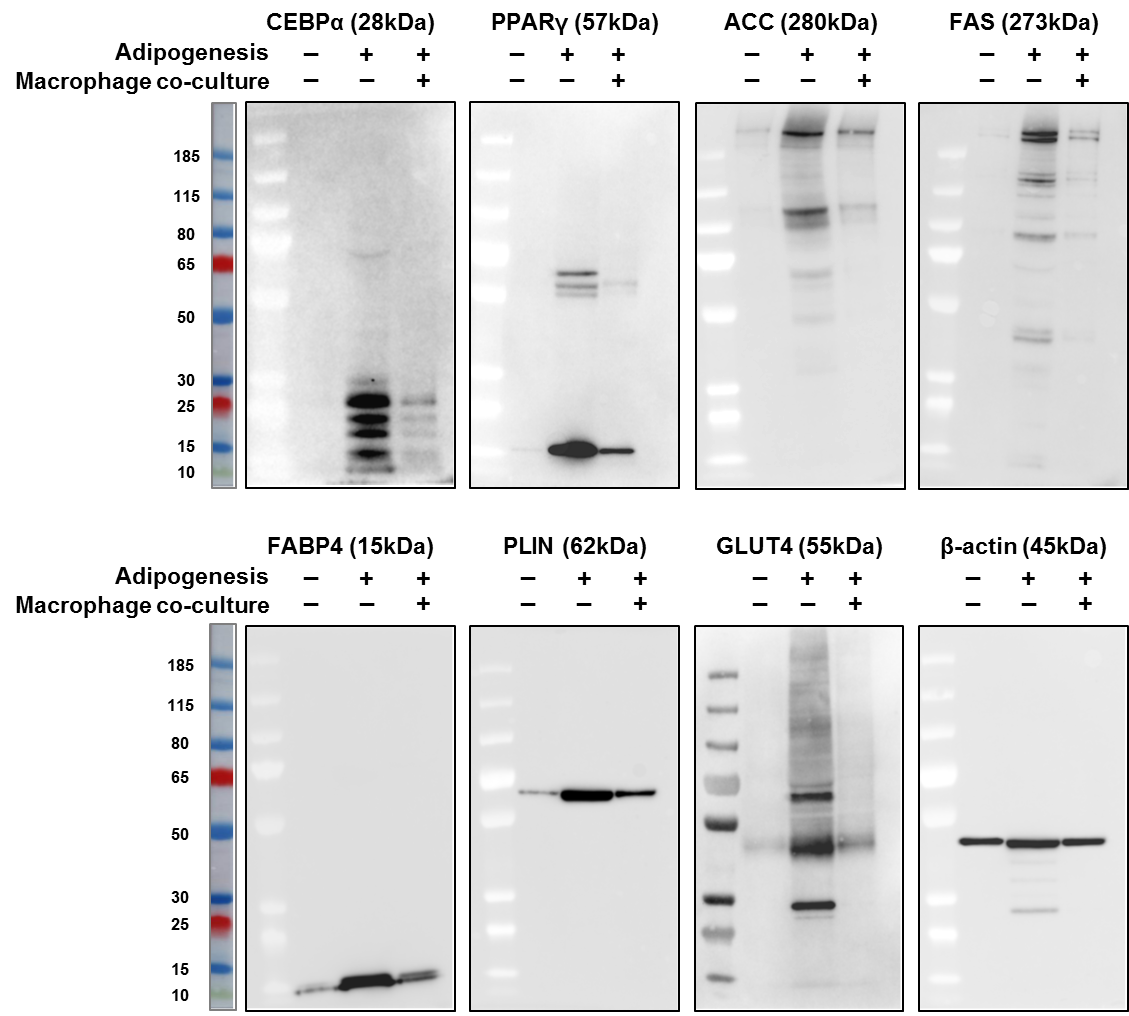


**Figure S1.** Full-length blots corresponding to Fig.1B in the main text.

**Supporting Figure S2**

**
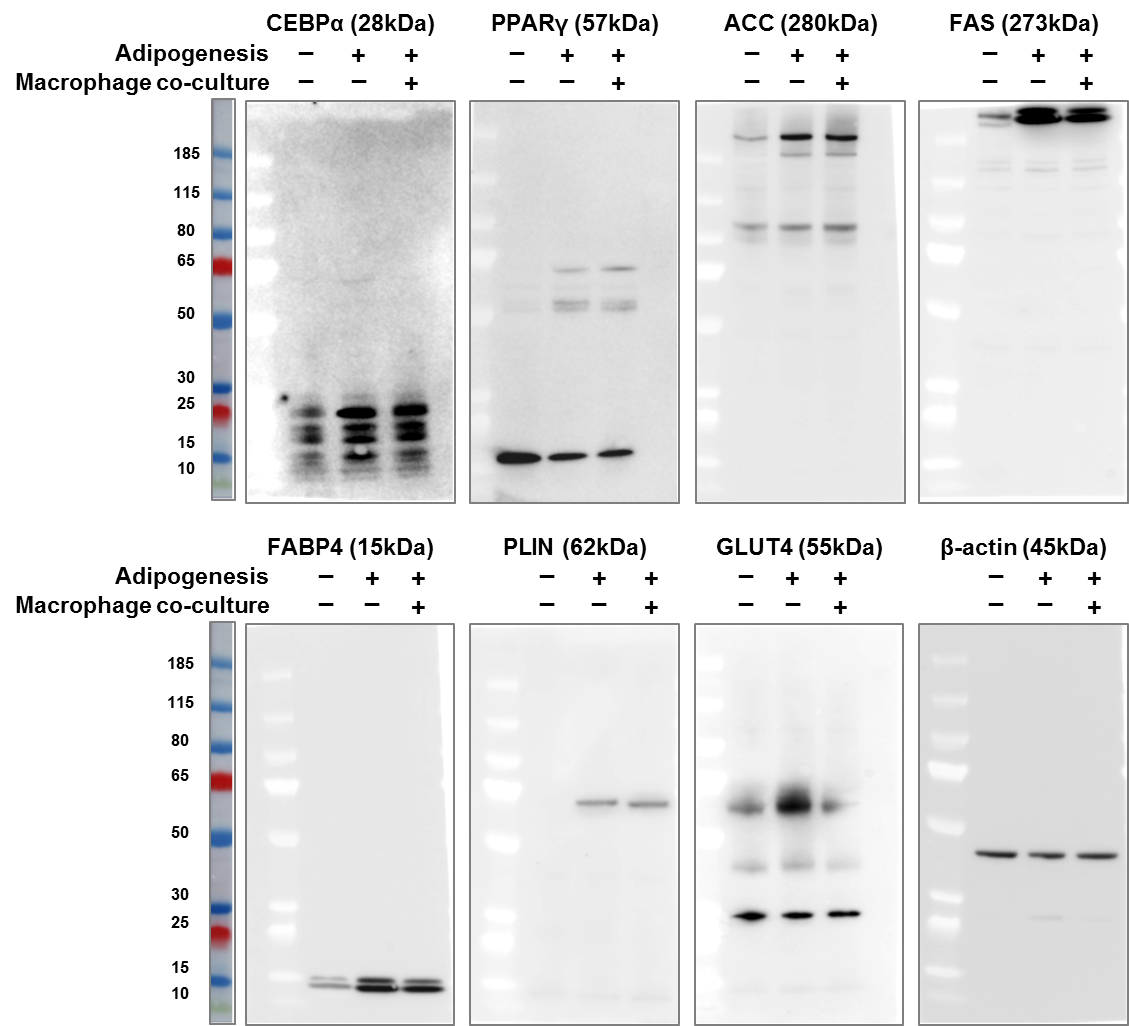
**

**Figure S2.** Full-length blots corresponding to Fig.1C in the main text.

**Supporting Figure S3**

**
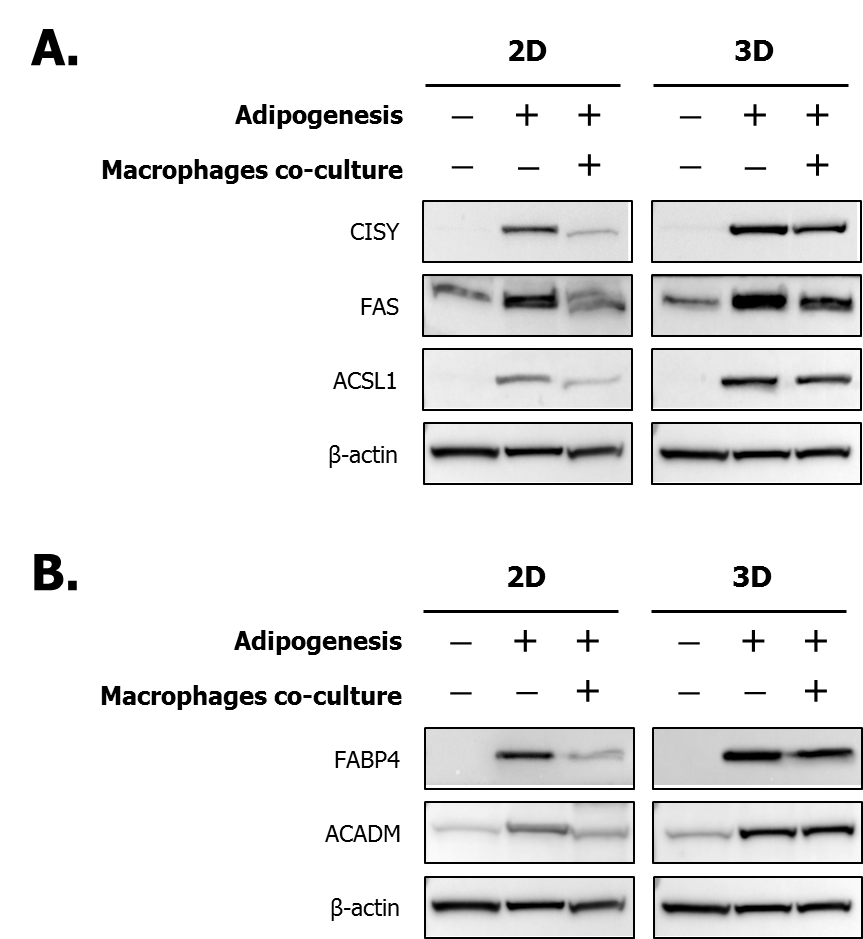
**

**Figure S3.** Validation of differentially expressed proteins associated with carbohydrate and fatty acid metabolisms in 2D and 3D culture system using western blot analysis. (A) The differential expressions of citrate synthase (CISY), fatty acid synthase (FAS), long-chain-fatty-acid--CoA ligase 1 (ACSL1) and a loading control (b-actin) associated to carbohydrate metabolism, and (B) the differential expressions of fatty acid binding protein 4 (FABP4), acyl-CoA dehydrogenase medium chain (ACADM) and a loading control associated to fatty acid metabolism from six different 3T3-L1 cells in the 2D and 3D cell-cultured systems. Full-length blots are presented in Figure S3-1 in Supporting Information.

**Supporting Figure S3-1**


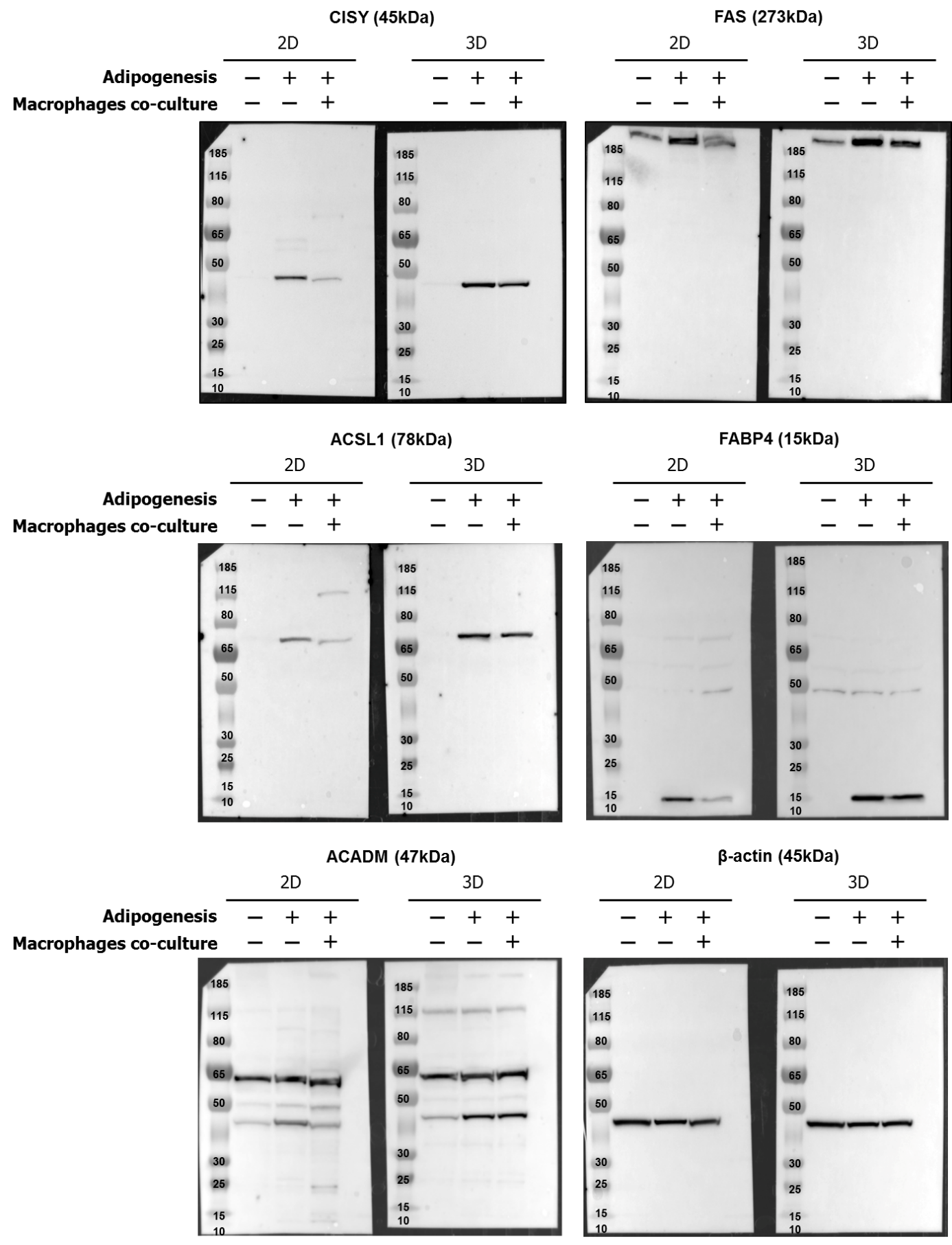


**Figure S3-1.** Full-length blots corresponding to Fig.S3 in Supporting Information.

**Supporting Figure S4**

**
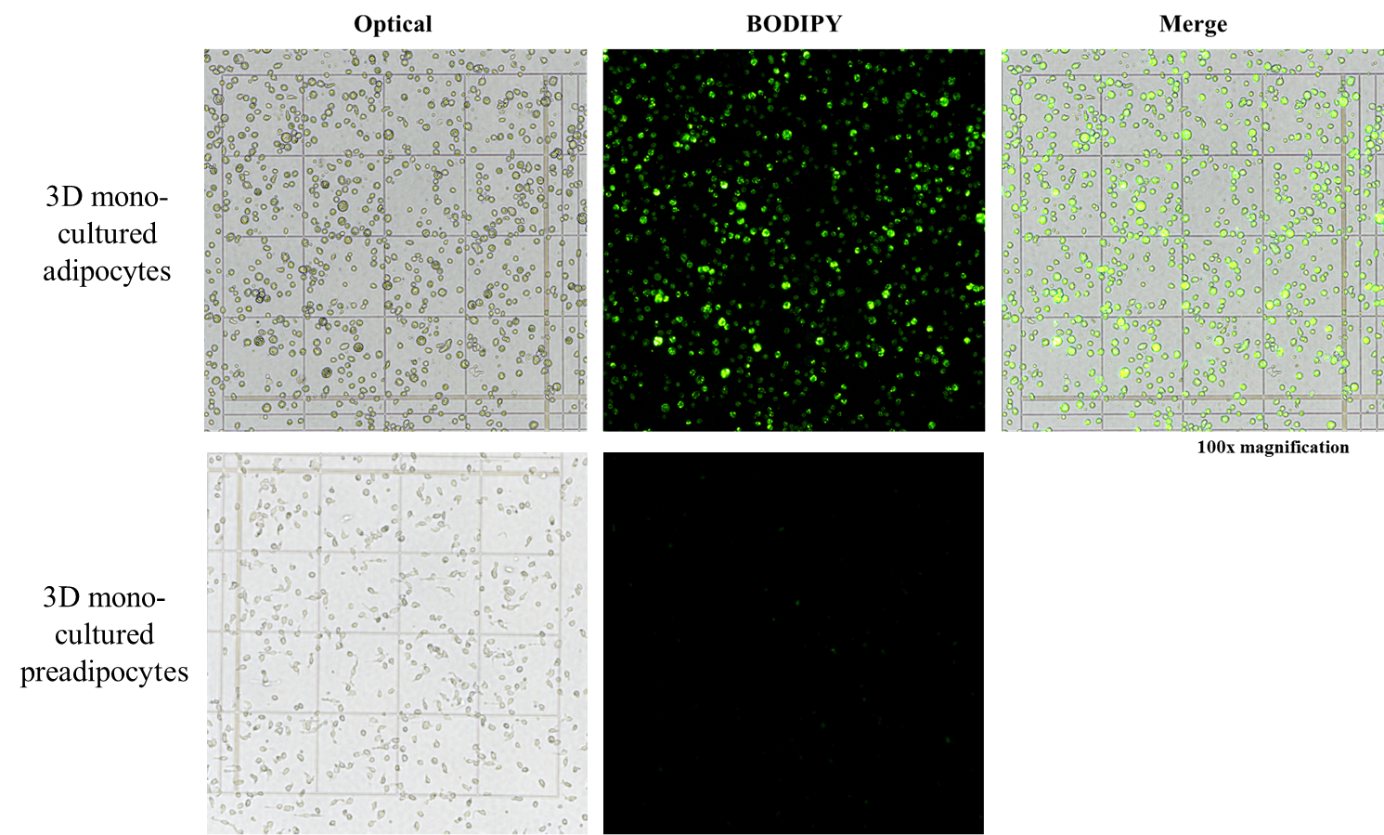
**

**Figure S4.** Lipid droplet fluorescent images of the 3D mono-cultured adipocytes (upper) differentiated from 3D mono-cultured preadipocytes (down)

**Supporting Figure S5**

**A**


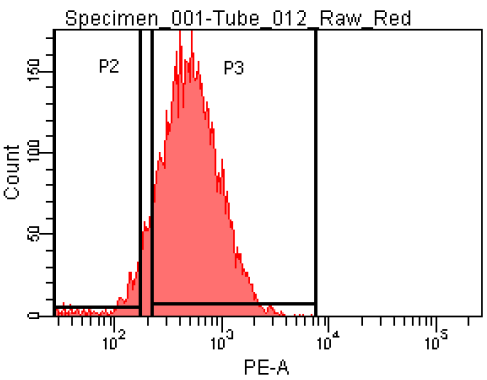


**B**


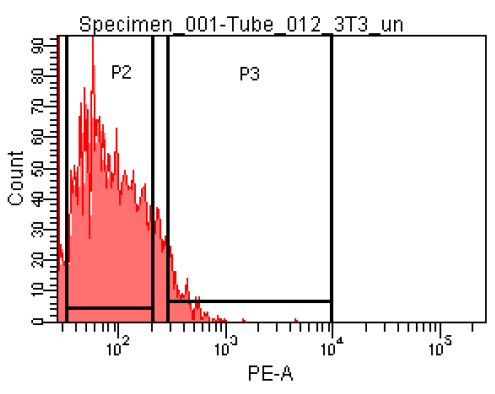


**24.0%**

**2.4%**

**Figure S5.** FACS analysis of 3T3-L1 adipocytes and red dye (Cell tracker) stained macrophages (Raw 264.7 cells) were co-cultured for 8 days. (A) cell tracking red dye positive control of Raw 264.7 (red), (B) red dye positive cells were determined by flow cytometry. The percentage of macrophages are measured to be about 10% of the total number of cells in 3D co-culture model.

**Supporting Figure S5-1**

**
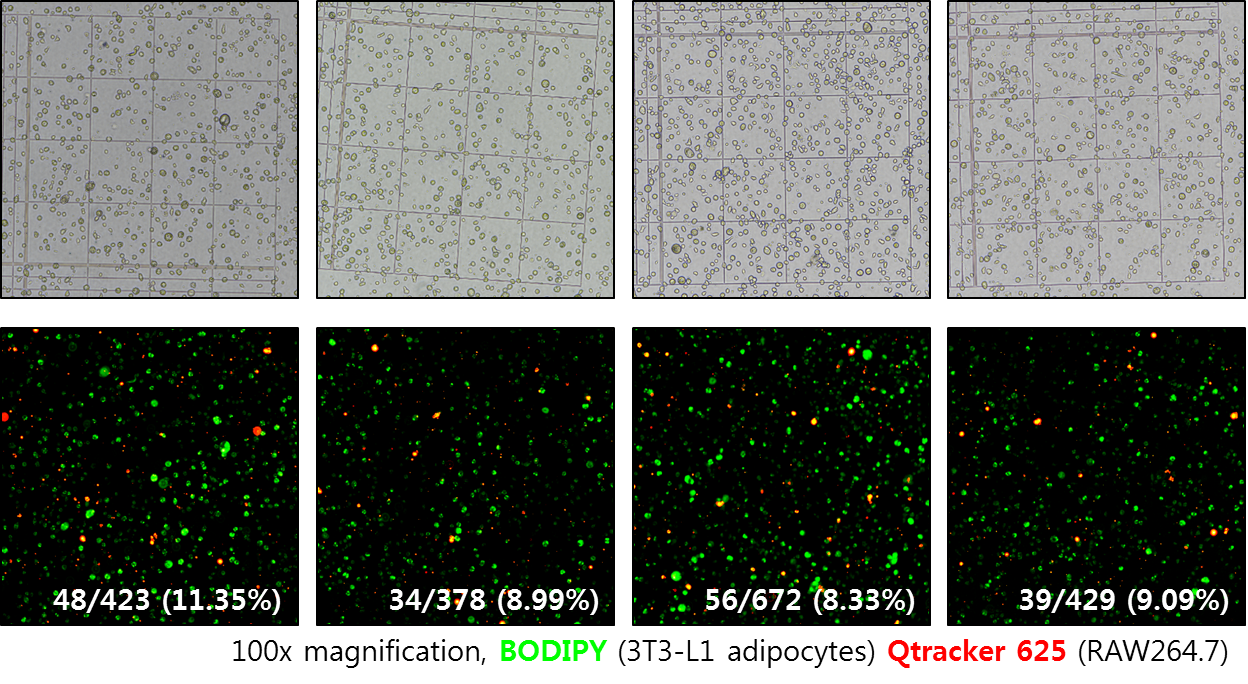
**

**Figure S5-1.** Lipid droplet fluorescent images of the adipocytes 3D co-cultured with macrophages. The percentage of macrophages are measured to be about 9.4% of the total number of cells in 3D co-culture model.

**Supporting Figure S6**

**
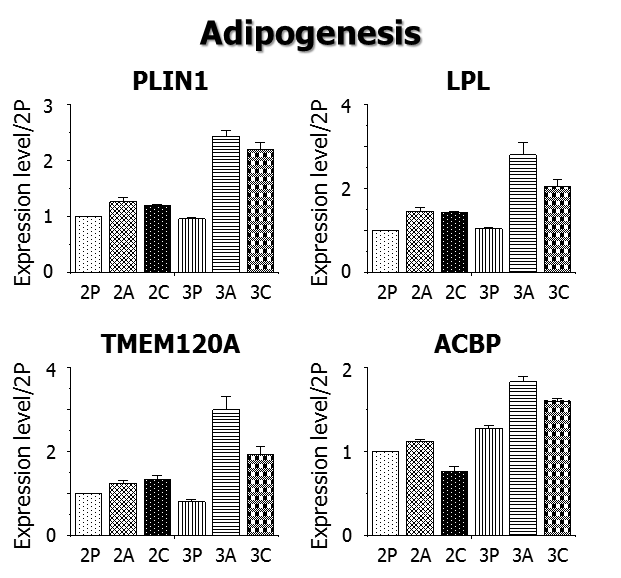
**

**Figure S6.** Expression levels of iTRAQ-labeled proteins associated to adipogenesis from six different 3T3-L1 cells cultured in the 2D and 3D systems. Protein expression levels were obtained by normalizing with 2D mono-cultured preadipocytes. Values are expressed as means ± S.E.M. (*Abbreviations: PLIN1, perilipin-1; LPL, lipoprotein lipase; TMEM120A, transmembrane protein 120A; ACBP, acyl-CoA-binding protein).

**Supporting Figure S7**

**
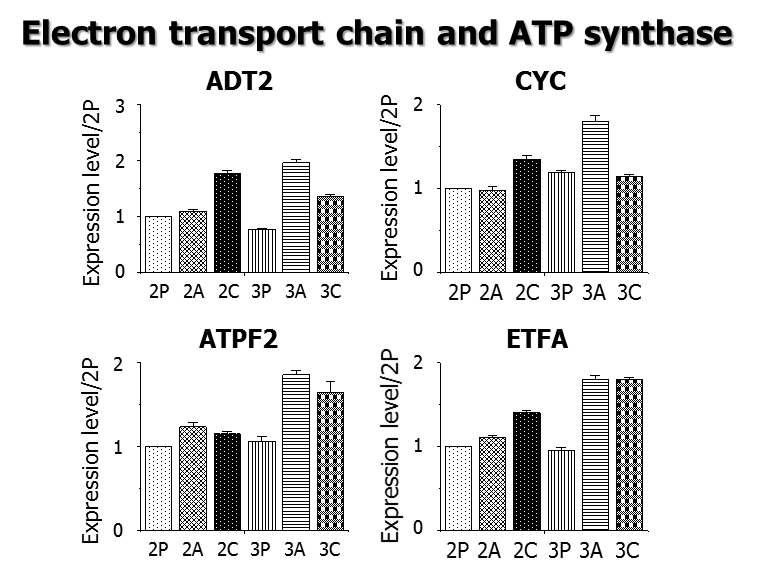
**

**Figure S7.** Expression levels of four different iTRAQ-labeled proteins related to electron transport chain and ATP synthase from six different 3T3-L1 cells cultured in the 2D and 3D systems. Protein expression levels were obtained by normalizing with 2D mono-cultured preadipocytes. Values are expressed as means ± S.E.M. (*Abbreviations: ADT2, ADP/ATP translocase 2; CYC, cytochrome c; ATPF2, ATP synthase mitochondrial F1 complex assembly factor 2; ETFA, electron transfer flavoprotein subunit alpha).

**Supporting Figure S8**

**
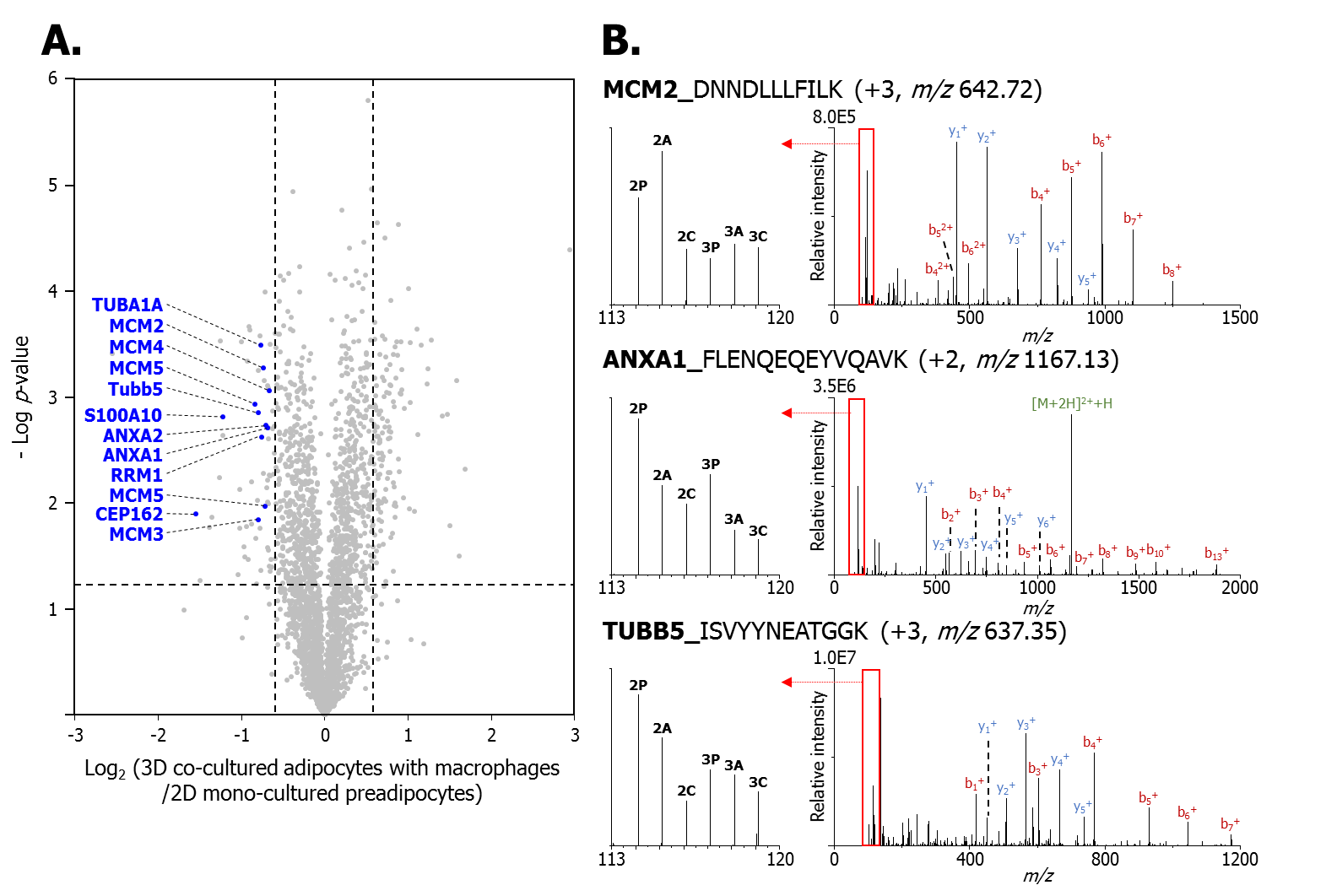
**

**Figure S8.** Volcano plot and MS/MS spectra of down-regulated proteins expressed in 3D co-cultured adipocytes with macrophages (3C) versus 2D mono-cultured preadipocytes (2P). (A) Volcano plot of quantified proteins between 3C and 2P illustrates from log_2_ 3C/2P iTRAQ ratio (x-axis) and –log p-value (y-axis). The non-axial vertical lines indicate ±1.5-fold change, and the non-axial horizontal line indicates *p* = 0.05, which is the statistical threshold for measuring the differentially expressed proteins. The 12 proteins denoted in blue among 66 down-regulated proteins were included in the cluster. (B) MS/MS spectra of MCM2 (DNNDLLLFILK), ANXA1 (FLENQEQEYVQAVK) and TUBB5 (ISVYYNEATGGK) are involved in cellular component organization, DNA replication and cell cycle. Left spectra indicate the iTRAQ reporter ions. 2P, 114.11; 2D mono-cultured adipocyte (2A), 115.11; 2D co-cultured adipocytes with macrophages (2C), 116.11; 3D mono-cultured preadipocytes (3P), 117.11; 3D mono-cultured adipocyte (3A), 118.11; 3C, 119.11.

**Supporting Figure S9**

**
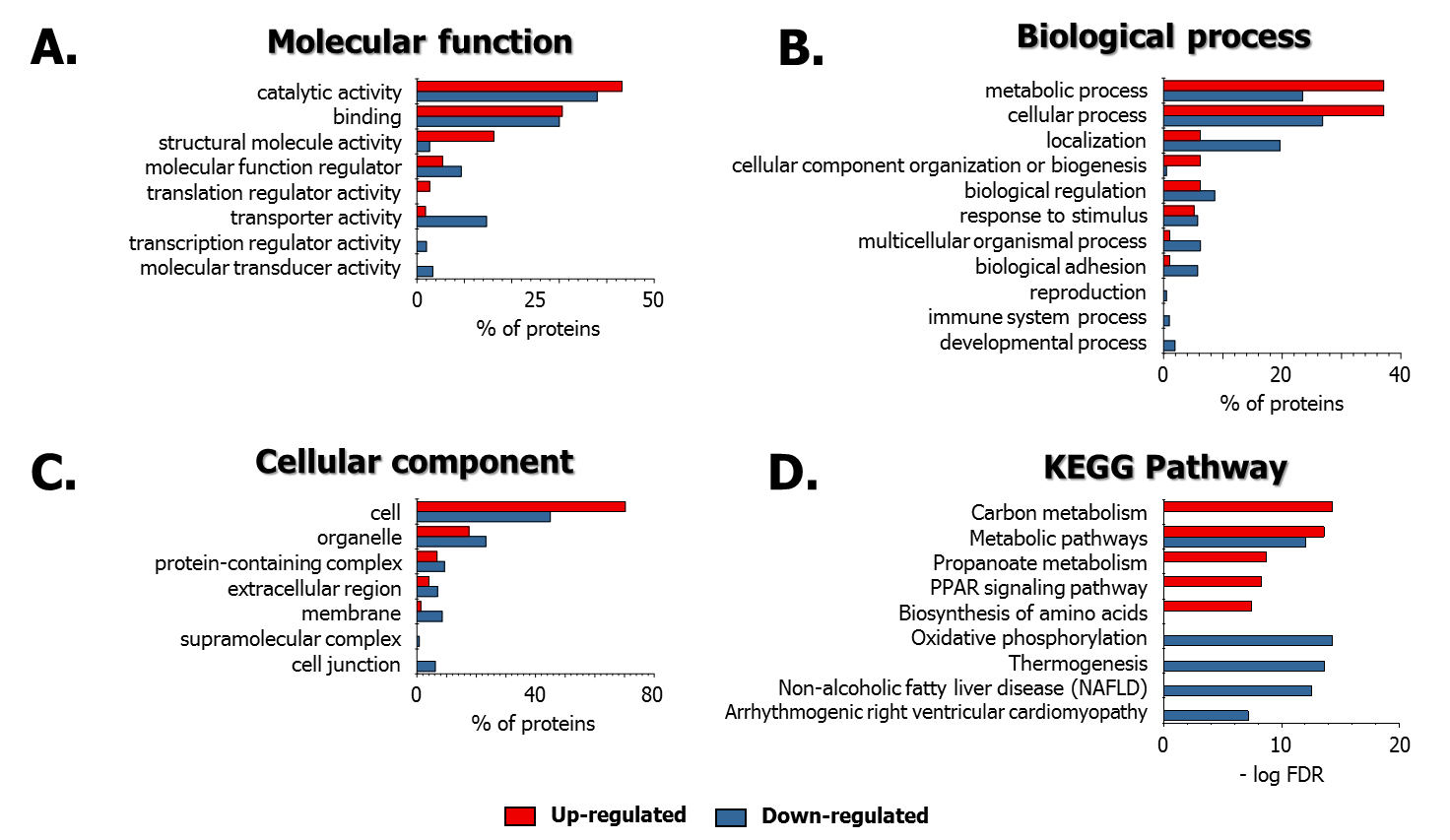
**

**Figure S9.** Functional annotation of up-regulated and down-regulated proteins in 3D co-cultured adipocytes with macrophages compared to those in 2D ones using the PANTHER classification system and STRING. (A) molecular function, (B) biological process, (C) cellular component, (D) KEGG pathway. For the classification of quantified proteins, 134 up-regulated and 220 down-regulated proteins from a total of 2,885 quantified proteins were used. The red and blue rods show the percentage (%) and –log FDR value of up- and down-regulated proteins, respectively. The KEGG pathways for the up-regulated and down-regulated proteins are listed in Supporting Table 2.

**Supporting Figure S10**

**
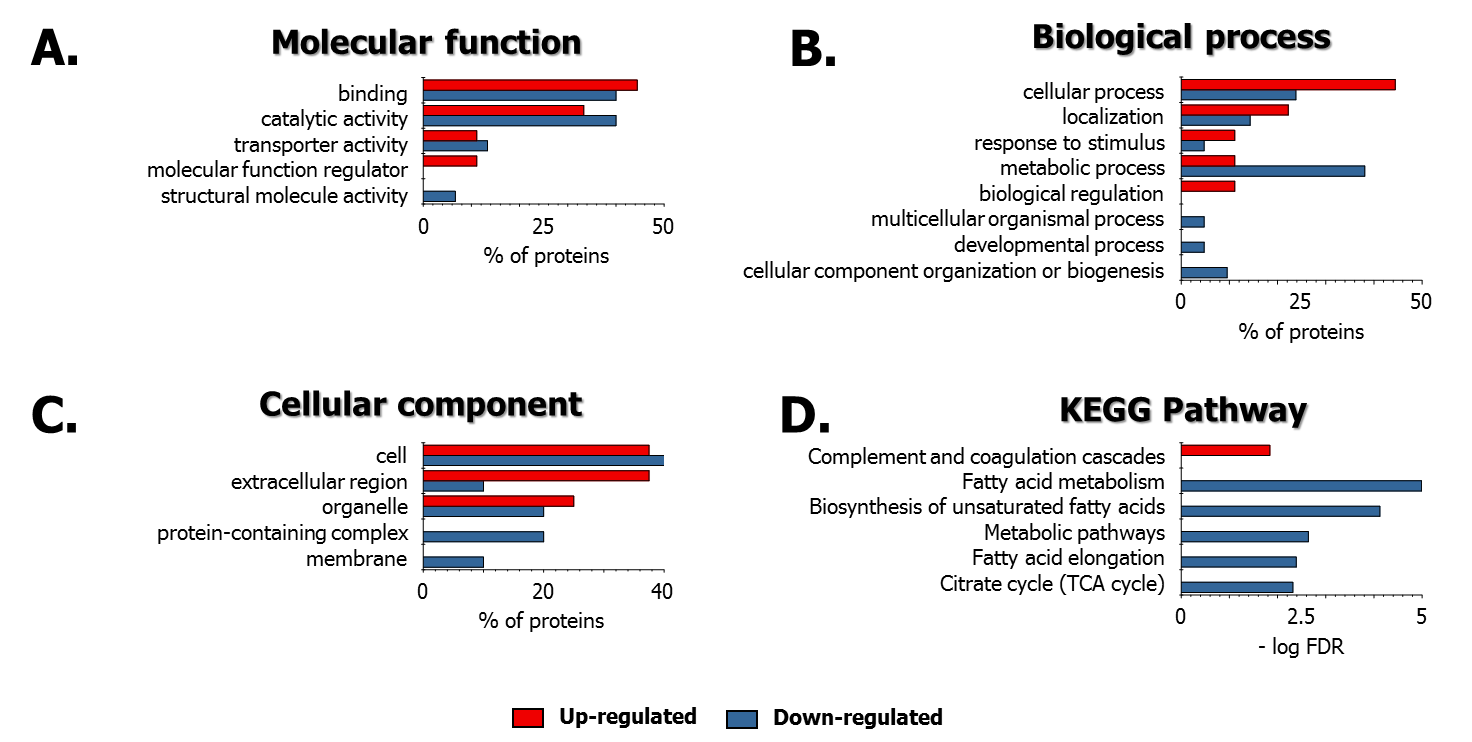
**

**Figure S10.** Functional annotation of up-regulated and down-regulated proteins in 3D co-cultured adipocytes with macrophages compared to those in 3D mono-cultured adipocytes using the PANTHER classification system and STRING. (A) molecular function, (B) biological process, (C) cellular component, (D) KEGG pathway. For classification of quantified proteins, 11 up-regulated and 22 down-regulated proteins from a total of 2,885 quantified proteins were used. The red and blue rods show the percentage (%) and –log FDR value of up- and down-regulated proteins, respectively. The KEGG pathways for the up-regulated and down-regulated proteins are listed in Supporting Table 2.

**Supporting Table**

**Table S1.** Lists of up-regulated proteins included in the network analysis in 3D co-cultured adipocytes with macrophages.

| **Up-regulated proteins (48)** | | |
| --- | --- | --- |
| **Abbreviation** | **Accession** | **Description** |
| **DECR1** | Q9CQ62 | 2,4-dienoyl-CoA reductase, mitochondrial |
| **AGT** | Q3UTR7 | Angiotensinogen |
| **PSAP** | E9PZ00 | Prosaposin |
| **FABP5** | Q05816 | Fatty acid-binding protein 5 |
| **C3** | A0A087WQH8 | Probable UDP-sugar transporter protein SLC35A4 |
| **GM2A** | Q60648 | Ganglioside GM2 activator |
| **ERO1L** | Q8R180 | ERO1-like protein alpha |
| **P4HA1** | Q60715 | Prolyl 4-hydroxylase subunit alpha-1 |
| **P4HA2** | Q5SX75 | Procollagen-proline, 2-oxoglutarate 4-dioxygenase (Proline 4-hydroxylase), alpha II polypeptide, isoform CRA_f |
| **P4HB** | P09103 | Protein disulfide-isomerase |
| **F5** | O88783 | Coagulation factor V |
| **CLU** | Q06890 | Clusterin |
| **ALDOA** | A6ZI44 | Fructose-bisphosphate aldolase |
| **HP** | Q61646 | Haptoglobin |
| **ALDOC** | P05063 | Fructose-bisphosphate aldolase C |
| **GAPDH** | P16858 | Glyceraldehyde-3-phosphate dehydrogenase |
| **PGK1-RS7** | P09411 | Phosphoglycerate kinase 1 |
| **PGAM1** | Q9DBJ1 | Phosphoglycerate mutase 1 |
| **TALDO1** | Q93092 | Transaldolase |
| **GPI1** | P06745 | Glucose-6-phosphate isomerase |
| **H6PD** | Q8CFX1 | GDH/6PGL endoplasmic bifunctional protein |
| **HK2** | O08528 | Hexokinase-2 |
| **LDHA** | Q564E2 | L-lactate dehydrogenase |
| **BCAT2** | O35855 | Branched-chain-amino-acid aminotransferase, mitochondrial |
| **BCKDHB** | Q6P3A8 | 2-oxoisovalerate dehydrogenase subunit beta, mitochondrial |
| **DLAT** | Q8BMF4 | Dihydrolipoyllysine-residue acetyltransferase component of pyruvate dehydrogenase complex, mitochondrial |
| **PDHA1** | P35486 | Pyruvate dehydrogenase E1 component subunit alpha, somatic form, mitochondrial |
| **DID** | O08749 | Dihydrolipoyl dehydrogenase, mitochondrial |
| **MPST** | Q99J99 | 3-mercaptopyruvate sulfurtransferase |
| **TST** | P52196 | Thiosulfate sulfurtransferase |
| **GOT2** | P05202 | Aspartate aminotransferase, mitochondrial |
| **MDH1** | P14152 | Malate dehydrogenase, cytoplasmic |
| **MDH2** | P08249 | Malate dehydrogenase, mitochondrial |
| **FH1** | P97807 | Fumarate hydratase, mitochondrial |
| **GLUD1** | P26443 | Glutamate dehydrogenase 1, mitochondrial |
| **SOD2** | P09671 | Superoxide dismutase [Mn], mitochondrial |
| **IDH3A** | Q9D6R2 | Isocitrate dehydrogenase [NAD] subunit alpha, mitochondrial |
| **IDH3B** | Q91VA7 | Isocitrate dehydrogenase [NAD] subunit, mitochondrial |
| **ACO2** | Q99KI0 | Aconitate hydratase, mitochondrial |
| **ETFA** | Q99LC5 | Electron transfer flavoprotein subunit alpha, mitochondrial |
| **VLCAD** | P50544 | Very long-chain specific acyl-CoA dehydrogenase, mitochondrial |
| **ACADM** | P45952 | Medium-chain specific acyl-CoA dehydrogenase, mitochondrial |
| **ECHDC1** | Q9D9V3 | Ethylmalonyl-CoA decarboxylase |
| **ALDH6A1** | Q9EQ20 | Methylmalonate-semialdehyde dehydrogenase [acylating], mitochondrial |
| **HADH** | Q61425 | Hydroxyacyl-coenzyme A dehydrogenase, mitochondrial |
| **HIBADH** | Q99L13 | 3-hydroxyisobutyrate dehydrogenase, mitochondrial |

**Table S1.** (Continued)

| **Abbreviation** | **Accession** | **Description** |
| --- | --- | --- |
| **SCAD** | Q07417 | Short-chain specific acyl-CoA dehydrogenase, mitochondrial |
| **HIBCH** | Q8QZS1 | 3-hydroxyisobutyryl-CoA hydrolase, mitochondrial |

**Table S2.** Lists of down-regulated proteins included in the network analysis in 3D co-cultured adipocytes with macrophages.

| **Down-regulated proteins (12)** | | |
| --- | --- | --- |
| **Abbreviation** | **Accession** | **Description** |
| **ANXA1** | P10107 | Annexin A1 |
| **ANXA2** | P07356 | Annexin A2 |
| **S100A10** | P08207 | Protein S100-A10 |
| **TUBB5** | P99024 | Tubulin beta-5 chain |
| **TUBA1A** | P68369 | Tubulin alpha-1A chain |
| **CEP162** | Q6ZQ06 | Centrosomal protein of 162 kDa |
| **RRM1** | P07742 | Ribonucleoside-diphosphate reductase large subunit |
| **MCM2** | P97310 | DNA replication licensing factor MCM2 |
| **MCM3** | P25206 | DNA replication licensing factor MCM3 |
| **MCM4** | P49717 | DNA replication licensing factor MCM4 |
| **MCM5** | P49718 | DNA replication licensing factor MCM5 |
| **MCM6** | P97311 | DNA replication licensing factor MCM6 |

**Supporting Methods.**

**Method S1.** Materials and chemicals

4-(2-Hydroxyethyl)-1-peperazineethanesulfonic acid (HEPES), potassium acetate, magnesium acetate, ammonium bicarbonate (ABC), DL-dithiothreitol (DTT), formic acid (FA), iodoacetamide (IAA), L-cysteine, triethylammonium bicarbonate (TEAB), bovine serum albumin (BSA), dimethyl sulfoxide (DMSO), insulin, isobutylmethylxanthine, and dexamethasone were purchased from Sigma (St. Louis, MO, USA). For cell culture in 2D and 3D system, Dulbecco’s modified Eagle’s medium (DMEM), Penicillin, and streptomycin were obtained from Gibco/Invitrogen (Carlsbad, CA, USA). Fetal bovine serum (FBS) and Hank’s Balanced Salt Solution (HBSS) were purchased from Thermoscientific (Carlsbad, MA). N-dodecyl beta-D-maltoside for the cell lysis and Coomassie protein assay reagent kit for the concentration of extracted proteins from 3T3-L1 cells were purchased from Thermo Scientific (Rockford, IL, USA). Protease inhibitor cocktail (EDTA-free, mini-tablet) was purchased from Biotool (Houston, TX, USA). Sequencing grade trypsin was obtained from Promega Corp. (Madison, WI, USA). Acetonitrile (ACN) and HPLC grade water from Burdick & Jackson (Ulsan, Korea) were used for a binary nLC separation of tryptic digests. Hydrophilic-lipophilic balance (HLB) cartridges (1 cc, 10 mg, and 1 cc, 30 mg) were purchased from Waters Corporation (Milford, MA, USA). The iTRAQ labeling reagent (8-plex) for MS-based quantitative analysis of the diverse 3T3-L1 cells was purchased from AB Sciex (Framingham, MA, USA)

**Method S2.** Lipid droplet fluorescent staining.

To distinguish between the differential rates of adipocytes and preadipocytes, the staining of mature adipocyte lipid droplets in the 3D printed scaffolds was performed using BODIPY 493/503 (Invitrogen, Carlsbad, CA, USA). The BODIPY 493/503 stock solution (2 mg/mL) was prepared in dimethyl sulfoxide (DMSO) and diluted 1:2000 (1 µg/mL BODIPY) in Hank’s Balanced Salt Solution (HBSS) for staining. For fixation, scaffolds were washed in HBSS and then incubated in 4% paraformaldehyde solution for 30 min at 4°C. Next, the paraformaldehyde solution was removed by three washes with HBSS, and the scaffolds were stained with 1 ml of diluted BODIPY solution for 30 min at 37°C. The stained lipid droplets in the scaffold were washed twice with HBSS and observed using a fluorescence microscope. For the purpose of measuring the ratios of macrophages to the total number of cells in the 3D co-culture model, the macrophage tracking in the 3D co-cultured scaffolds was also determined using the Qtracker® 625 Cell Labelling Kit (Thermo, Carlsbad, CA, USA) and observed using fluorescence microscopy (Eclipse TE2000-U, Nikon, Tokyo, Japan). 3T3-L1 preadipocytes and stained RAW264.7 macrophages with tracking dye (1% to 10% concentration, vol./vol.) were seeded with hydrogel mixture. Thereafter, the hydrogel mixture was fabricated to a 3D scaffold. After an 8-day differentiation period, scaffolds were lysed and trypsinised by 10 mM EDTA and then cells were counted using a disposable haemocytometer-based cell counter (SKC Co. Ltd., Seoul, Korea) with the aid of an inverted microscope (Eclipse TE2000-U, Nikon, Tokyo, Japan). Fluorescence microscopy images were taken under the same circumstances and conditions.

**Method S3.** Fluorescence-activated cell sorting analysis

To measure the ratios of macrophages to the total number of cells in the 3D co-culture model, 3T3-L1 and red dye (Cell tracker)-stained Raw 264.7 cells were co-cultured for 8 days. After co-culture, these cells were washed with DPBS containing 2.5% FBS and centrifuged at 1800 rpm for 5 min. Collected cells were visualised by fluorescence-activated cell sorting (FACSAria, BD Biosciences, Belgium) and analysed with BD FACSDiva software.

**Method S4.** Online 2D-nanoLC-ESI-MS/MS for shotgun analysis of 2D- and 3D-mono/co-cultured adipocyte proteome.

We analysed the resulting digests using a coupled Q-Exactive^TM^ hybrid quadrupole orbitrap tandem mass spectrometer (Thermo Scientific, Bremen, Germany) with a model 1260 capillary LC system (Agilent Technologies, Waldbronn, Germany) via an electrospray ionisation (ESI) source. The trapping columns and analytical column were prepared as described in our previous studies.^60,61^ Tryptic digests from six different 3T3-L1 cells retained on 1-dimensional [4 cm length of capillary (100 μm-i.d., 360 μm-o.d.) packed 1 cm of C18 resins (3 µm, 200Å, Bonna-Agela Technologies Inc.)] or 2-dimensional [4 cm length of capillary (200 μm-i.d., 360 μm-o.d.) packed 0.5 cm of C18 resins (5 µm, 200Å, Prontosil) and then continuous packed 2.0 cm of SCX resins (5 µm, 200Å, Waters Corporation)] trapping column were introduced into an analytical column [15 cm length of capillary (75 μm-i.d., 360 μm-o.d.) packed C18 resins (3 µm, 100Å, Bonna-Agela Technologies Inc.)]. The tryptic peptides were eluted with the following binary gradient of mobile phases A (0.1% FA in water) and B [0.1% FA in 98/2 (v/v) ACN/water]: 2% B for 10 min, 2% to 8% B for 0.5 min, 8% to 15% B for 4.5 min, 15% to 30% B for 70 min, 30% to 90% B for 3 min, 90% B for 15 min, 90% to 2% B for 2 min, and then maintained for 15 min to re-equilibrate the analytical column. The ESI of the eluted peptides was conducted in the analytical column at a flow rate of 200 nL/min by controlling the length of the capillary tube (25 μm-i.d., 360 μm-o.d.) that was assembled to the micro-tee for splitting the flow from the HPLC pump. Sample fractionation using a SCX-RP trap column was carried out with a total of 20 steps [0, 10, 15, 20, 22, 24, 25, 26, 27, 28, 29, 30, 35, 40, 45, 50, 75, 200 and 1,000 mM of ABC buffers in 0.1% FA solution, and 1,000 mM of ABC buffer in NH_4_OH solution (pH 10)] by injecting 10 µL of buffer solution. The MS scan (*m/z* 300–1800) was performed in the positive ion mode setting to an ESI voltage of 2.5 kV, a capillary temperature of 270 °C, and a target value of 1e6. For comprehensive profiling and quantification of peptides from 2D and 3D 3T3-L1 cells, the MS/MS parameters utilising a high collision dissociation (HCD) were set to a normalised collision energy (NCE) of 27% and a dynamic exclusion duration of 20 s. The mass resolution (R_mass_) and injection time (t_max_) were set to 70,000 and 80 ms for full MS, and 17,500 and 60 ms for targeted MS/MS, respectively.
